# Population viability analysis of the endangered Dupont’s Lark *Chersophilus duponti* in Spain

**DOI:** 10.1038/s41598-021-99125-y

**Published:** 2021-10-07

**Authors:** Alexander García-Antón, Juan Traba

**Affiliations:** 1 Terrestrial Ecology Group (TEG-UAM), Department of Ecology, Universidad Autónoma de Madrid, C/ Darwin, 2., 28049 Madrid, Spain; 2 Centro de Investigación en Biodiversidad Y Cambio Global, Universidad Autónoma de Madrid (CIBC-UAM), C/ Darwin, 2., 28049 Madrid, Spain

**Keywords:** Conservation biology, Ecological modelling

## Abstract

Steppe lands in Europe are critically affected by habitat loss and fragmentation, and hold over 50% of IUCN Red List bird species in Europe. Dupont’s Lark is a threatened steppe-specialist passerine whose European geographic range is restricted to Spain, with less than 2000 pairs and an annual population decline of − 3.9%. Its strongly fragmented habitat leads to a metapopulation structure in the Iberian Peninsula that includes 24 populations and 100 subpopulations. We present an updated Population Viability Analysis based on the latest scientific knowledge regarding distribution, population trends, breeding biology and connectivity. Our results predict metapopulation extinction in 2–3 decades, through a centripetal contraction process from the periphery to the core. The probability of extinction in 20 years was 84.2%, which supports its relisting to Endangered in Spain following IUCN criteria. We carried out a sensitivity analysis showing that some parameters, especially productivity and survival of adults and juveniles, help to increase metapopulation viability. Simulation of management scenarios showed that habitat restoration in a subset of key subpopulations had a positive effect on the overall metapopulation persistence. Translocations of a limited number of individuals from source to recipient locations may help to rescue the most endangered subpopulations without reducing the global time to extinction of the metapopulation. In addition, we identified the most critical areas for action, where local populations of the species are prone to extinction. This work suggests that the viability of the Dupont’s Lark metapopulation could be improved and its risk of extinction reduced if urgent and localized conservation measures are applied. In the short-term, habitat loss and fragmentation due to ploughing, reforestation and infrastructures implementation in Dupont’s Lark habitat must be avoided. Habitat restoration and translocations could help to avoid imminent extinction of critical subpopulations. Restoration of extensive grazing is recommended as the most effective way to achieve the long-term conservation of Dupont’s Lark in Spain.

## Introduction

Habitat loss is currently a main concern for biodiversity conservation and its impact is expected to increase this century^1^. Most of the Earth’s surface has been altered from its natural conditions (in 2016, only 23.2% of the world’s terrestrial surface was considered wilderness^2^) and global biodiversity conservation must also address the preservation of biodiversity in anthropic landscapes^3^. In addition to direct habitat loss, deterioration of habitat quality increases risks associated with landscape fragmentation, related to the disappearance of optimal habitat and to a greater isolation of populations^4^. This can be especially dramatic for rare species or for those inhabiting narrowly distributed habitats^5^. In fragmented populations, the landscape matrix and the species dispersal capacity will determine the movement of individuals among populations and the maintenance of gene flow^4^, necessary for the long-term persistence of small and strongly isolated populations^6^ and the maintenance of viable population sizes^7^. Additionally, genetic stochasticity caused by inbreeding and genetic drift can affect fragmented populations, especially the smallest ones^8^. In summary, fragmentation increases isolation, triggering inbreeding problems, which can negatively impact non-migratory species with short dispersal movements^3,5^. All of these factors affect numerous species of conservation interest, especially birds associated with treeless environments and natural steppes, which are very rare in Europe^9–15^.

In fragmented distributions, a species can form the same population if the isolated habitat patches are connected through individual movements allowing gene flow between fragments, forming a metapopulation. Classic metapopulation theories include Levins colonization-extinction balance^16^ and source-sink patterns defined by Hanski^17^, although these theoretical approaches seldom fit wild populations. Both classic models assume the relevance of patch size and connectivity: small and isolated patches have a lower probability of presence and higher probability of extinction than large and connected ones^16,17^. Habitat quality also plays a relevant role in patch occupancy, affecting population viability and persistence^18,19^.

Steppes and agrarian pseudo-steppes (grass-steppes, sensu Traba et al*.*^20^) are crucial habitats for bird conservation, harboring 55% of European bird species listed in the IUCN Red List^21,22^. Overall, more than 80% of steppe bird species show an unfavorable conservation status in Europe^21,23^ (e.g., Little Bustard^9^, Stone-curlew^10^, Northern Wheatear^11^ or Ortolan Bunting^12^) with a continuous negative trend in both population size and distribution range. This is a consequence of the accelerated process of land use changes faced by steppe and steppe-like habitats, with dramatic consequences on steppe bird populations across Europe^21–25^.

The main drivers of steppe-bird population decline in Europe are related to: (i) land use changes, such as afforestation, new crops or infrastructure development^21,26^; and (ii) agricultural intensification, both at the landscape configuration level (landscape homogenization, irrigation, changes in agriculture and livestock management) and at the plot level (increased use of machinery and agrochemicals)^21,24,25–30^. One of the main effects of agricultural intensification is the reduction in spatial heterogeneity, at both field and landscape scales^24^. In addition, the disappearance of traditional land uses practices such as extensive grazing has negative effects on steppe habitat quality^31^, because this abandonment facilitates tree regeneration^32^, alters invertebrate species composition^33^ and limits arthropod availability^34^.

Spain is the stronghold for steppe-birds in Western Europe, harboring a large proportion of their total European present-day breeding population^21^. However, most of Spanish steppe bird populations declined during the 1990–2000 period^21^ and later^35^. A species of particular conservation concern is the Dupont’s Lark, whose populations declined by around 40% during the period 2004–2015^36^. Dupont’s Lark (*Chersophilus duponti*, Vieillot 1820) is a scarce and threatened alaudid whose European distribution is restricted to Spain^37^. The Iberian population is isolated from the African ones^38^ and there is scarce gene flow within the Spanish metapopulation^39^, which predicts high extinction risk especially for the smallest and most isolated subpopulations^40^. Its strong dependence on flat, low-shrub natural steppes^39–47^, and the extreme fragmentation of its optimal habitat in Spain, has driven the species to a fragmented metapopulation distribution, with a surface of occupation of ~ 1000 km^2^ in Spain^48^ and the existence of adequate, unoccupied habitat patches that would act as stepping stones^48,49^. Although dispersive movements of the species remain unknown, recent work suggests the existence of medium distance movements to some extent, but insufficient to connect the most peripheral and isolated subpopulations^49^. The European population size has been estimated to be less than 4000 males^50^ with an extremely male-biased sex ratio^51,52^, which clearly reduces the effective population size.

Dupont’s Lark is included in the Birds Directive (79/409/CEE) Annex I (species and sub-species that are particularly threatened) and was recently listed as Vulnerable in the World on the IUCN Red List. In Spain it is listed as Vulnerable in the National Catalogue of Threatened Species (R.D. 139/2011). The current population decline in Spain was estimated at an average of − 3.9% annually, with greater declines experienced by peripheral, small populations^36^. This decline has been attributed to progressive habitat loss by natural succession promoted by rural abandonment, the development of infrastructures within the species’ habitat (mainly wind farms), and land use changes such as ploughings or reforestations^26,53,54^, all of which exacerbate fragmentation and isolation. In addition, a decrease in traditional extensive sheep grazing modifies habitat quality^55,56^ where habitat remains. If no change occurs in public policies (mainly, in the Common Agricultural Policy), this trend will be maintained or accelerated in the near future, considering the high number of projects planned in the areas of the species’ occurrence, especially windfarms^57^, and the trend in agrarian subsidies^58^. Furthermore, the species seems sensitive to climate change, as increases in temperature range and decreases in annual precipitation are associated with probability of extinction^48^. Despite this dramatic scenario, management measures have been carried out with success^56^ and could be implemented in critical populations to slow down the population trend. In addition, translocation of individuals could promote gene flow and help to avoid the extinction of the most endangered populations^50^.

In this work, we performed a complete analysis of the viability of the Iberian metapopulation of Dupont’s Lark, in order to: (i) establish an overall assessment of extinction risk and the parameters to which it is most sensitive; (ii) identify critical populations where management measures are urgently needed; and (iii) evaluate the effect of habitat management measures on local and global extinction risk, including habitat restoration and translocation of wild individuals. Finally, we evaluated the importance of isolation and climate in the risk of extinction of the whole metapopulation.

Population viability analysis (PVA) is an essential tool in wildlife conservation, that helps estimate population decline and extinction risk^59,60^, identifying critical areas in a fragmented population^61^, evaluating and prioritizing management frameworks^62^ and finding knowledge gaps to be addressed by future research^63^. Thus, the results of this work can provide useful support for management decisions and rapid application for the conservation of this threatened species as well on the rest of the coexistent bird community in the Iberian natural steppes.

## Results

### Base model

In our baseline model with 500 iterations, we obtained a mean growth rate (*r*) of − 0.401 (95% CI [− 0.423, − 0.380]) for the Spanish metapopulation. This decline generated a predicted population size of 311.52 (95% CI [294.122, 328.918]) individuals in 10 years with 0% probability of extinction, and 8.06 (95% CI [7.524, 8.596]) with 84.2% probability in 20 years (Supplementary Figure S1). The mean (*T*_*mean*_) and maximum (*T*_*max*_*)* times to extinction were 19.07 (95% CI [18.85, 19.29]) and 27 years respectively. In the most favorable scenario (maximum time to extinction, meaning all 500 iterations result in no extant subpopulations) the metapopulation would survive 27 years. 18% of the subpopulations would become extinct during the first 10 years, 62% during the second decade and 20% would remain extant during the third decade (Supplementary Table S1).

Subpopulations’ extinction occurred radially, showing a progressive range contraction from the most peripheral subpopulations to the metapopulation core (Supplementary File S1). During the first decade the model predicted a strong decline in population size with a 100% persistence probability of the metapopulation, with a steep decline in persistence probability after year 12 (Supplementary Figure S1).

### Critical areas

Nineteen subpopulations became extinct in the first two years (*T*_*mean*_ < 2, Supplementary Table S2). These subpopulations had low initial population sizes (mean *N*_*0*_ = 1.29; min. = 0; max. = 4 individuals) and a mean linear distance to the metapopulation core of 451.72 km (min. = 42.4, max. = 449.1 km). Those subpopulations overcoming a *T*_*mean*_ of 10 years (n = 29, Supplementary Table S1) had higher initial population sizes (mean *N*_*0*_ = 156.90; min. = 1; max. = 1,125 individuals) and were located closer to the metapopulation centroid (mean = 79.47, min. = 10.2, max. = 154.1 km)*.* The Pearson's test showed a significant correlation between *T*_*mean*_ and *N*_*0*_ (r = 0.46, p < 0.001), and *T*_*mean*_ and the distance to the metapopulation centroid (r = -0.44, p < 0.001).

According to population decline rates, two thirds of the subpopulations (69%) showed negative values of population growth (*r*). Among the subpopulations with a stronger decline were those with higher initial population size and located in the core of the distribution (see Supplementary Table S2 for a summary of the 10 subpopulations with the strongest annual decline, and Supplementary Table S1 for the complete list).

### Sensitivity analysis

**Figure 1:**
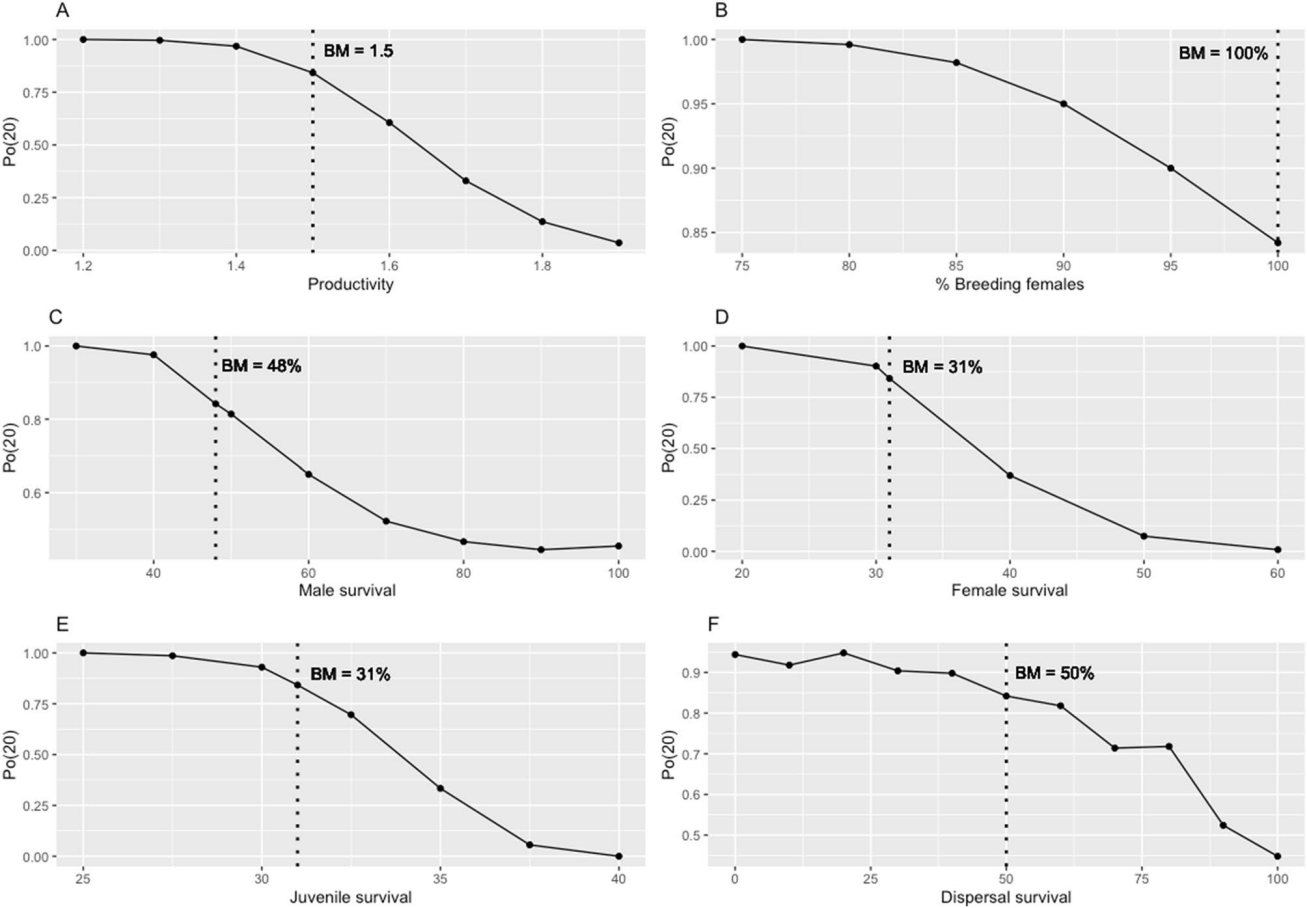
Sensitivity of the probability of metapopulation extinction in 20 years, *P*_*0*_*(20)*, to the different parameters evaluated. Dotted lines represent the value used in the base model (BM). (**A**) Productivity (offspring/brood); (**B**) % of breeding females; (**C**) Adult male survival; (**D**) Adult female survival; (**E**) Juvenile survival (both sexes); (**F**) Survival of dispersers. See effects on *r*, *T*_*mean*_ and *T*_*max*_ in Supplementary Table S3 and Supplementary Figs. S2–S4.

All the parameters evaluated markedly affected the metapopulation viability. A range in productivity from 1.2 to 1.9 offspring/clutch produced a variation in probability of extinction in 20 years from 1 to 0, respectively, with a stronger effect for the intermediate values of 1.4–1.7 (Fig. 1A). In the base model with a value of productivity of 1.5 offspring/brood we obtained a probability of extinction in 20 years of 84.2%. An increase to 1.6 offspring/brood showed a reduction of that probability to 60.6%, while a reduction to 1.4 offspring/brood would increase the probability of extinction to 96.8%. The effects on *r*, *T*_*mean*_, and *T*_*max*_ were similar to *P*_*0*_*(20)* (see Supplementary Table S3 and Supplementary Figs. S2–S4).

In relation to the percentage of breeding females, the metapopulation showed a high sensitivity. Our base value was of 100% breeding females due to the species sex ratio, obtaining 84.2% probability of extinction in 20 years. A reduction to 90% breeding females predicted an increase in that probability to 95%, and reaching the threshold of 75% breeding females would drive the species to extinction in 20 years (Fig. 1B).

The metapopulation showed a similar sensitivity to reductions in survival of the different sexes and age classes. We used the values of 48% adult male survival, and 31% for females and juveniles, obtaining 84.2% probability of extinction. Reaching the lowest thresholds of 30% (adult males), 20% (adult females) and 25% (juveniles) predicted a 100% probability of metapopulation extinction in 20 years (Fig. 1C–E). The effect of increasing survival rates differed among classes. Increasing male survival had a positive effect reducing extinction probability, though always above 0% (*P*_*0*_*(20)* = 45.4% with a 100% survival of adult males; Fig. 1C, Supplementary Table S3), while increases in female and juvenile survival (respectively, thresholds of 60% and 40%) avoided metapopulation extinction in 20 years (Fig. 1D–E).

Finally, according to dispersal survival, we used a value of 50% in the base model to obtain a 84.2% probability of extinction in 20 years. Without dispersal survival, the model predicted a probability of extinction of 99.4%, while a survival of 100% would result in a *P*_*0*_*(20)* of 44.8% (Fig. 1F, Supplementary Table S3).

### Habitat restoration

The simulation of habitat management had a positive effect on the metapopulation overall values of *T*_*mean*_, *r*, *P*_*0*_*(20)* and *T*_*max*_ (Supplementary Tables S4, S5). All scenarios of habitat management increased the viability of the metapopulation compared to the base model.

**Figure 2:**
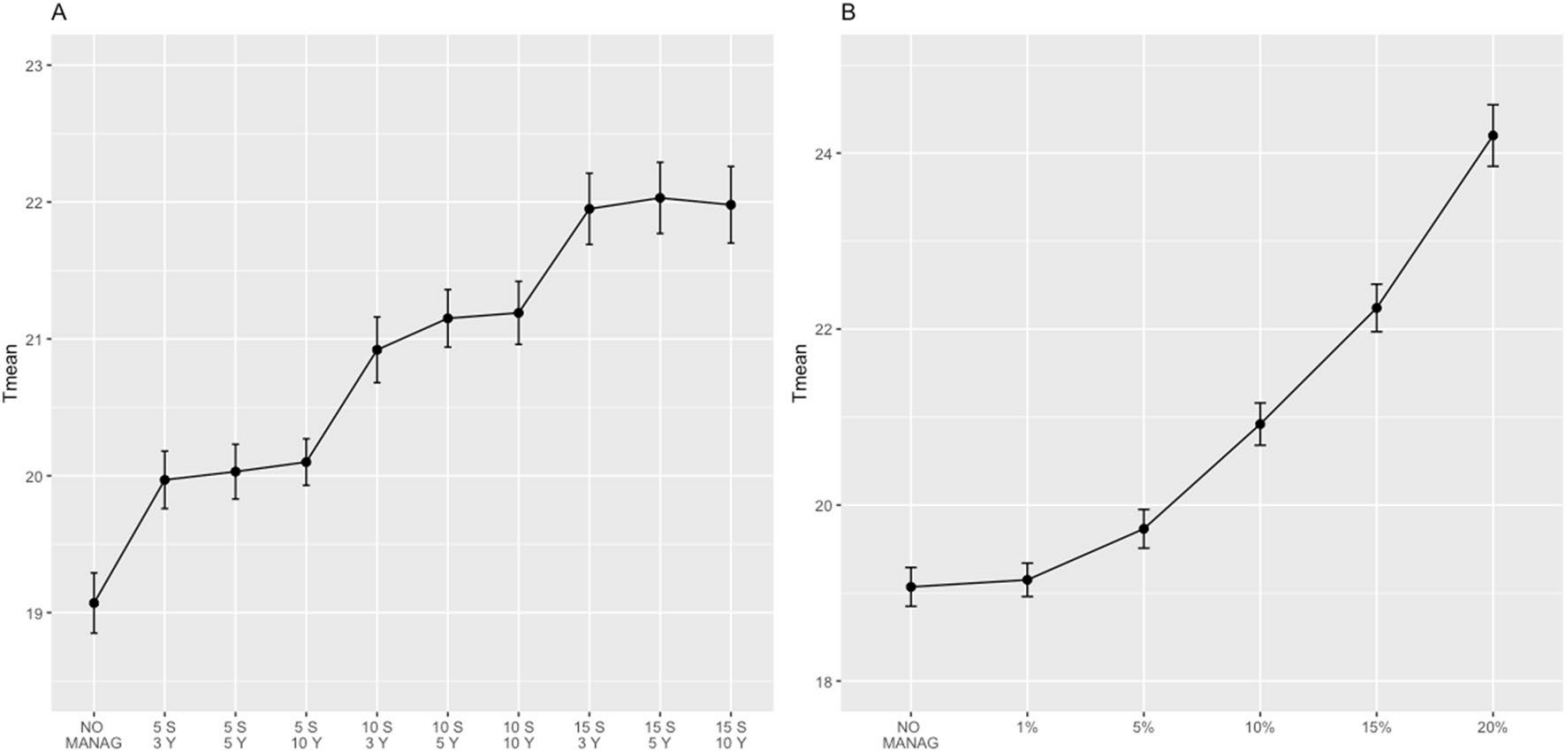
Effects of habitat restoration simulations in metapopulation mean time to extinction. (**A**) nine different scenarios varying the number of subpopulations managed (selecting those with higher population size: 5, 10 and 15 subpopulations) and time period of the program (3, 5 and 10 consecutive years). (**B**) using the alternative of 10 subpopulations during 3 years of management (considered of real applicability), five scenarios of management intensity were tested (1, 5, 10, 15 and 20% increase of productivity and carrying capacity).

Mean time to extinction increased with the number of subpopulations and years of management (Fig. 2A). Two-way ANOVA showed a stronger effect of increasing the number of subpopulations (*F*(3, 4391) = 225.328, *p* < 0.001) than the time period (*F*(2,1) = 0.077, *p* = 0.926), and the Tukey’s post hoc test showed significant differences (p < 0.001) among all levels of the factor (0, 5, 10 and 15 subpopulations managed). The intensity of management increased the metapopulation mean time to extinction (Fig. 2B). One-way ANOVA showed a significant effect (*F*(5, 18,416) = 617.2, p < 0.001) and the post hoc test revealed differences between all levels: no management, 1, 5, 10, 15 and 20% increase in productivity and K.

Similarly, habitat restoration simulations increased metapopulation growth rate (Supplementary Figure S5), reduced the probability of extinction in 20 years (Supplementary Figure S6) and increased the maximum time to extinction (Supplementary Figure S7).

### Translocation

**Figure 3:**
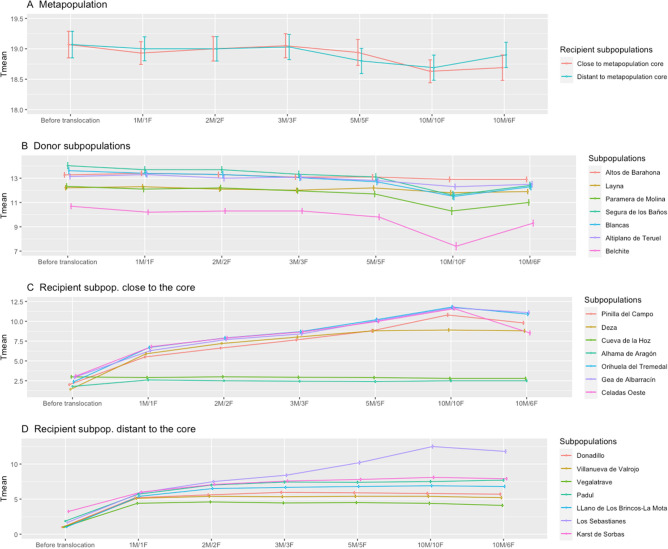
Effects in mean time to extinction (*T*_*mean*_ and 95% confidence interval) in the metapopulation, donor and recipient subpopulations after the translocation program. Donor subpopulations were those with more than 100 males (n = 7). Recipient subpopulations were those with the most unfavorable situation in the PVA results (shortest mean time to extinction), two scenarios were simulated: seven recipient subpopulations close to the metapopulation core and seven recipients distant from the core. For each scenario, six different harvest alternatives are offered: 1 + 1, 2 + 2, 3 + 3, 5 + 5, 10 + 10 and 10 + 6 males/females, this last one adjusted to the species sex ratio. In all of them, movements were carried out during 3 consecutive years. Harvested individuals were introduced randomly in recipient subpopulations (Supplementary Tables S6, S7).

The translocation of individuals produced a slight decrease in the overall metapopulation mean time to extinction (Fig. 3A). Two-way ANOVA showed a significant effect of harvesting (F(6, 138) = 4.372, p < 0.001). The post-hoc Tukey test revealed a significant difference with the 'before translocation' scenario appeared when harvesting 10 males and 10 females (diff. = − 0.414, p = 0.001). The rest of the harvesting levels showed no significant differences with the 'before translocation' scenario.

Harvesting also showed a significant effect in donor subpopulations (*F*(5, 7118) = 199.6, *p* < 0.001), which in general reduced their mean time to extinction (Fig. 3B). Significant differences with the 'before translocation' scenario appeared in all harvesting scenarios except 1 M/1F (diff. =  − 0.119, p = 0.516 and 10 M/6F (diff. =  − 0.036, p = 0.995). Translocations showed a positive effect in recipient subpopulations close to (F(5, 71,042) = 2279, p < 0.001) and distant from (Fig. 3D; F(5, 89,213) = 4938, p < 0.001) the metapopulation core (Fig. 3C,D respectively). The post-hoc test showed that differences occurred between the ‘before translocation’ scenario and all harvesting alternatives (all of them with p < 0.001).

The rest of the parameters (growth rate, probability of extinction and maximum time to extinction) showed similar results (Supplementary Figures S8, S9 and S10 respectively; Supplementary Table S6).

### Factors affecting population viability

The GLM showed a significant influence of isolation and climate on metapopulation persistence (Table 1). In the first case, the distance to the metapopulation centroid had a negative effect on the mean time to extinction (Table 1), meaning shorter extinction times as the subpopulation is further from the core, supporting the aforementioned range contraction process. According to climate, the model revealed shorter times to extinction with increases in annual precipitation trends (Table 1), suggesting that subpopulations with increases in aridity along the period 2000–2018 could persist over more years.

**Table 1:** Summary of the GLM coefficients to evaluate the effect of geographic and climatic variables on the mean time to extinction (*T*_*mean*_) of the metapopulation.

	Estimate	Std. Error	t value	p value
(Intercept)	− 4.778e−17	0.086	0.000	1.000
Dist. C	− 0.493	0.083	− 5.583	< 0.001
Annual P. trend	− 0.275	0.083	− 3.116	< 0.01

Variables initially included were: distance to the metapopulation centroid (Dist. C), distance to the nearest subpopulation (Dist. N), minimum January (*T*_*jan*_) and maximum August (*T*_*aug*_) temperatures (mean values of the period 2000–2018), annual precipitation (mean 2000–2018) and the trends of the three climatic variables (as the slope of a linear regression of the period 2000–2018). Only the significant variables remaining after sequential model simplification are shown (see details in text).

## Discussion

**Figure 4:**
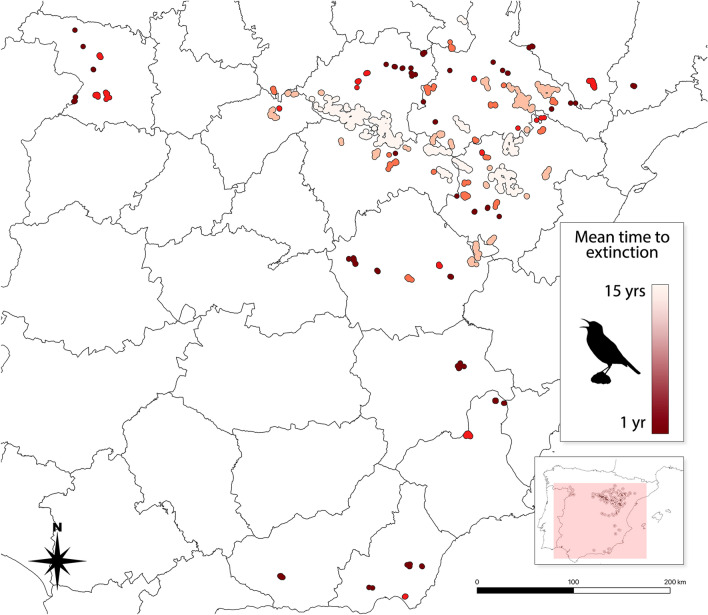
Mean time to extinction of the 100 subpopulations conforming Dupont’s Lark metapopulation in Spain. The PVA model predicts the extinction of the last subpopulations in around 15 years (center of the distribution). The peripheral subpopulations face imminent extinction (dark maroon color).

This work offers updated information on the viability of the Spanish metapopulation of the Dupont’s Lark Spanish metapopulation and highlights the dramatic conservation status of the species in Europe. In summary, our work suggests that, if there is no change in present-day driving forces, the species will reach extinction in a few decades, through a process of centripetal contraction from the periphery to the core subpopulations of the Iberian Range and Ebro Valley (Fig. 4, Supplementary File S1). However, the species is sensitive to management measures, especially to increases in productivity and survival of adults and juveniles, which offers opportunities for its conservation. Finally, subpopulations at high risk of extinction may be rescued via translocation of wild individuals from source subpopulations.

Our model shows that small and more isolated subpopulations have a shorter mean time to extinction. Subpopulations facing an imminent extinction (*T*_*mean*_ < 2 years) according to the model predictions had extremely low population sizes and longer distances to the nearest subpopulation and metapopulation centroid (Supplementary Table S2). These patches are more vulnerable to stochastic demographic and environmental processes, as well as genetic drift^40,64^. The core areas (Layna, Altos de Barahona, Altiplano de Teruel, amongst others; Supplementary Table S2) showed longer times to extinction but also the most pronounced population decline (higher negative *r* values). An important fraction of the population inhabits small patches with a high risk of extinction, where local reproduction may be insufficient to compensate for mortality and whose persistence would rely on the immigration of individuals from nearby source patches^65^.

The available data reveals that recolonizations are an extremely rare event in Dupont’s Lark, and local extinctions seem to be irreversible. Our monitoring in the Iberian Range has revealed the gradual extinction of small patches with only one recolonization in the post-2000 period^26,36^. At a metapopulation scale, among the 23 subpopulations extinct since the year 2000 only one recolonization has been recorded^66^. Extinct or unoccupied areas may play an important role as stepping stones, which were included in the dispersion matrix of the PVA model, and could play a relevant role in the maintenance of the overall metapopulation, as they increase the connectivity among patches^49^. Our results showed that large and connected patches are crucial in the conservation of the Dupont’s Lark, which is in line with the conceptual framework of metapopulation theory. We may have expected higher recolonization and persistence probability^17^ in larger and connected patches^40,64^ however, our results do not support a Levins model of colonization-extinction balance since recolonizations are extremely rare events, while extinctions are permanent. The process seems to be a radial contraction of the distribution range towards the metapopulation core, probably associated with an increase in landscape fragmentation as the distance to the center increases in a source-sink pattern revealing a centripetal contraction process from the periphery to the core subpopulations of the Iberian Range and Ebro Valley^49^).

We suggest that the range contraction process, acting first on smaller and peripheral patches, is occurring both at local and metapopulation scales, which offers little chance for a patch to be reoccupied once it has gone extinct. This is not incompatible with recent findings of new settlements of the species (explained by the use of new monitoring methods^67^), which we attribute to previous insufficient sampling effort rather than true recolonizations.

Our results at the metapopulation level are similar to those obtained in a recent analysis of Dupont’s Lark occupancy at a smaller scale^55^, which detected a hierarchical pattern in habitat use, with metapopulation-scale factors (connectivity and patch size) explaining most of the variance versus landscape and microhabitat-scale variables. While genetics suggests scarce gene flow^39^ among Spanish subpopulations, individual movements could be occurring to some extent according to both the data available about juvenile dispersion^68^ and connectivity analysis^49^. Several efforts to analyze gene flow and structure in Dupont’s Lark have been made in the recent years^39,40^. However, the rapid extinction process of the species requires more genetic research to quantify effects of isolation and trends toward homozygosity, especially in small and peripheral populations.

Management and conservation measures should be guided to enhance three crucial aspects: interpatch dispersion, minimum patch size and habitat quality. We highlight the convenience of a two-level management approach, concordant with both types of critical areas (Supplementary Table S2). First, the core subpopulations showed strong declines, with high negative *r* values. In these areas, measures directed at maintaining or increasing habitat quality and carrying capacity should benefit productivity and slow down this decline. Second, the small and most isolated subpopulations must be protected against imminent extinction, to urgently reduce the contraction process of the metapopulation. Here, management should aim at guaranteeing a minimum patch size and interpatch connectivity. In this sense, connectivity analysis^49^ could help identifying key subpopulations with a potential effect as stepping stones and connectors for their neighbors.

As a general consideration, actions that improve productivity and adult and young survival should be implemented, at least, in the critical subpopulations. Our results showed that increasing the number of managed subpopulations offered better results than lengthening the time period of management (Supplementary Table S4, Fig. 2A). Predator control has been implemented as a useful and cost-effective tool to increase breeding success of ground nesters^69,70^ (but see also^69–73^) and could be evaluated for Dupont’s Lark. Food supplementation enhances population viability in endangered species^74,75^ and could be introduced as a short-term action in critical subpopulations. Particularly, sown sheep dung could simulate grazing in terms of coprophagous availability^76^. Habitat management showed a high potential for improving the viability of the metapopulation, as the action over a small subset of subpopulations had an effect on the whole metapopulation persistence (Fig. 2). Habitat restoration has been successfully carried out in Dupont’s Lark peripheral subpopulations successfully^50^ and may be implemented in the short term in critical areas, including shrub clearing and tree removal. In the medium term, the reintroduction of extensive grazing is the most recommended measure, as it preserves habitat quality and food availability in shrub steppes.

Translocations offer a good opportunity for management and should be explored as a useful tool for short-time rescue of small subpopulations at imminent extinction risk, but must be applied with caution, as the simulations showed that increasing the number of yearly harvested individuals up to 20 (10 males and 10 females) could promote a significant reduction in the metapopulation time to extinction. Moving a low number of individuals can balance the conservation of critical subpopulations with metapopulation persistence. Special attention must be paid to donor subpopulations, as the effects in the reduction of their time to extinction were significant in almost all scenarios of harvesting. The effects were slight up to 10 individuals (5 males, 5 females) harvested yearly (Fig. 3B), but with strong improvements in recipient subpopulations. Thus, this measure should be considered as a short-term way to reinforce subpopulations facing imminent extinction or rescue recently extinct ones. More research is needed regarding translocations, as they could play an important role in mitigating inbreeding and increasing allele diversity in such a fragmented population, considering that more than 50% of the Spanish subpopulations are below 5 individuals^50^ and gene flow is lacking^39^.

As a medium-to-long term objective, management actions should be considered only as part of a broader framework of self-viability. A reliable conservation strategy to ensure the persistence of Dupont’s Lark in Europe should include the restoration of extensive grazing in natural steppes^56^, as it shapes plant structure and provides food through its positive effect on arthropod availability^55,56^. Habitat loss and fragmentation, both due to abandonment or land use changes (ploughing, reforestation or infrastructure installation), must be urgently stopped to slow down species decline.

The results of modeling geographic and climate effects on the metapopulation persistence also highlighted the radial contraction process towards the core regions. Our PVA results forecast a future Dupont’s Lark distribution restricted to the Iberian Range and the Ebro Valley in about one decade if the process is not reverted (Fig. 4). The higher resistance to extinction of those subpopulations with a more pronounced trend toward aridity (Table 1) could suggest that this steppe species would benefit from a future climate change scenario, but this must be considered with caution as it may be associated with a geographic effect^77^. While steppes and other sub-desert systems could take advantage of a future increase in aridity (as some records support^78^), steppe bird species are usually strict specialists, as is the case of Dupont’s Lark, making them extremely susceptible to habitat changes, whatever the direction. Thus, more research about effects of future climate change scenario is needed in this sense.

Finally, according to the IUCN Red List Categories and Criteria (IUCN 2012), our results point to the necessity of updating the species status to Endangered in Spain, following the criterion E (quantitative analysis indicating the probability of extinction in the wild to be ≥ 20% in 20 years or 5 generations, whichever is longer). Also, management measures aimed at improving the conservation status of Dupont’s Lark may also benefit other declining steppe birds, such as Little bustard^9^, Stone curlew^10^, Northern wheatear^11^ or Ortolan bunting^12^, among others, which have experienced a general negative trend in recent years in Spain and Europe^13–15^, as well as the steppe habitat itself.

## Methods

The ethics committee of Animal Experimentation of the Autonomous University of Madrid as an institution enabled by the Community of Madrid (Resolution 24th September 2013) for the evaluation of projects based on the provisions of Royal Decree 53/2013, 1st February, has provided full approval for this research (CEI 80-1468-A229). All experiments were performed in accordance with relevant guidelines and regulations.

### Species and study area

The Dupont´s Lark is a relatively well monitored species. Broad scientific information has been produced in the last two decades regarding many aspects of its ecology and biology. Our study area includes the totality of the known European distribution range, which is restricted to continental Spain, currently comprising 24 populations and 100 subpopulations^49^. Criteria for the definition of these entities are based on the species’ movements^49^ and on the map of georeferenced locations and associated distance buffers. As a result, subpopulations are defined by clusters of observations separated by less than 5 km (considered resident movements) while populations are groups of subpopulations separated by a maximum distance of 20 km (considered the probable juvenile dispersive distance), see details in García-Antón et al*.*^49^.

**Figure 5:**
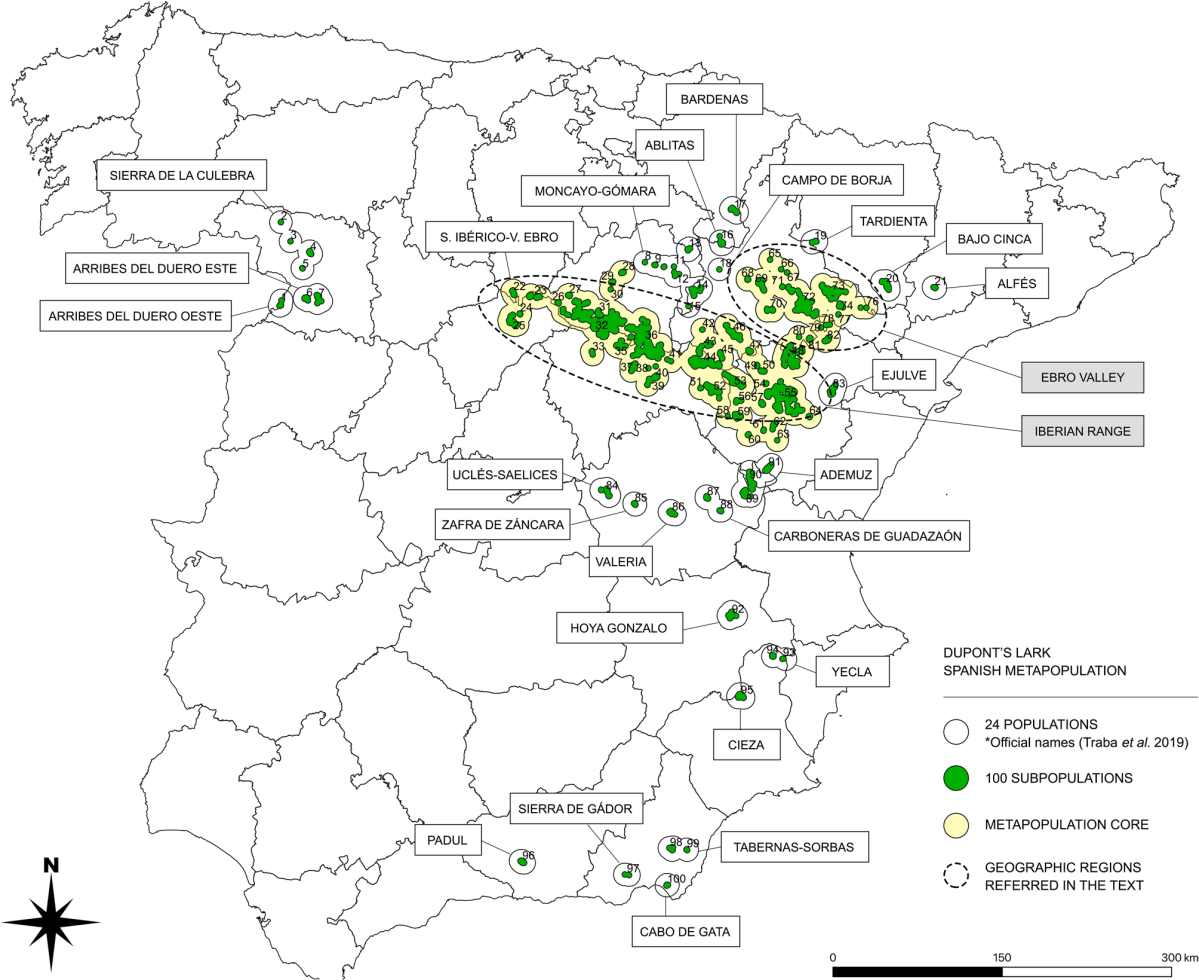
Dupont’s Lark Spanish metapopulation, comprising 24 populations (black contour) and 100 subpopulations (green). The core of the metapopulation (light yellow) is formed by two geographic regions (Iberian Range and Ebro Valley), merged in a large single population with the last definition criteria^49^.

The core of the metapopulation comprises two large geographic regions: the Iberian Range and the Ebro Valley (Fig. 5), which are merged into one, single population following the definition criteria. We consider as a starting point the metapopulation structure recently published in the National Conservation Strategy of the species^50^. Some of the 100 subpopulations considered have extremely low population sizes and are expected to reach extinction during the following years.

Risk of extinction has been previously assessed at a local scale in the Ebro Valley^79^ and in the Iberian Range^80^, and for the whole Spanish population^81^. However, new data have been published since then, and monitoring programs have increased capture-recapture data and updated the basics on distribution^48^, connectivity and dispersal^49,68^, breeding biology^82^ and population trends^36,83,84^. This allowed us to update critical parameters such as longevity and mortality, productivity, initial population size and carrying capacity, and re-evaluate the population viability of Dupont’s Lark at a national scale (Table 2). We used data from different monitoring programs carried out during the last 20 years, including the II National Census (2004–2007^85^) and from monitoring and research projects performed during the last 10 years, which included seasonal censuses, territory mapping, capture-recapture programs, radiotracking and record of behavioral data. We gathered 16,676 georeferenced locations of Dupont’s Lark from 2000 to 2018, including our own data and that from public administrations, other research institutions and private ornithologists (see also^48^).

**Table 2:** Values of the population parameters used to build the base model in VORTEX.

Parameter	This analysis	Source	Population	Sensitivity and alternative models	10% SD added to values
Inbreeding depression—lethal equivalents	6.29	Lacy and Pollak 2017			
Inbreeding depression—lethal alleles	50%	Lacy and Pollak 2017			
EV correlation among populations	50%	Suárez and Carriles 2010	Metapopulation		
Youngest age of dispersal	1 year	García-Antón et al*.* 2015	Iberian Range		
Oldest age of dispersal	1 year	Assumed			
Survival of dispersers	50%	Assumed		0–100%	
Dispersal rate among populations	10% dispersers and updated connectivity analysis	Laiolo et al*.* 2008, García-Antón et al*.* 2020	Ebro Valley, metapopulation		
Reproductive system	Monogamous	Authors’ own data			
Age of first offspring	1 year	Suggested (Suárez et al*.* 2009a)	Metapopulation		
Maximum lifespan	5 years	Authors’ own data	Iberian Range		
Maximum age of reproduction	5 years	Authors’ own data	Iberian Range		
Maximum number of broods per year	3	Pérez-Granados et al*.* 2017	Ademuz		
Maximum number of progeny per brood	5	Pérez-Granados et al*.* 2017	Ademuz		
Sex ratio at birth	50%	Suggested (Suárez et al*.* 2009b)	Metapopulation		
Adult females breeding	100%	Assumed		75–100%	X
Distribution of broods per year	6–29-65% females lay 1–2-3 broods, respectively	Estimated from Skylark (Suárez and Carriles 2010)			
Productivity (offspring/brood)	1.5	Pérez-Granados et al*.* 2017	Ademuz	1.2–1.9	X
Mortality of females age < 1 year	69%	Highest value available	Metapopulation	60–75%	X
Mortality of males age < 1 year	69%	Highest value available	Metapopulation	60–75%	X
Mortality of females age > 1 year	69%	Estimated from males (Suárez and Carriles 2010)	Metapopulation	40–80%	X
Mortality of males age > 1 year	52%	Mean of the 2 data points available (Laiolo et al*.* 2008, Vögeli et al. 2008)	Ebro Valley	0–70%	X
Catastrophes	1 (5% years, red. 5% breeding and survival	Suárez and Carriles 2010	Iberian Range		
Males in breeding pool	80%	Radiotracking (Suárez and Carriles 2010)	Iberian Range		
Initial population size	Most updated census available inferred to 2020 by population trend	Gómez-Catasús et al. 2018a	Metapopulation	See translocation program in text	
Carrying capacity	Density observed by radiotracking (1 ind./10 ha) * habitat surface and quality, with K truncation	Garza et al*.* 2005, Gómez-Catasús et al. 2018a, García-Antón et al*.* 2019	Iberian Range	See habitat restoration program in text	X

The base model was built considering the most plausible values given the available current information. Alternative scenarios and iterations were built to carry out the sensitivity analysis and simulations of translocation and habitat restoration programs (details in the text).

### Population viability analysis

We used the stochastic simulation software VORTEX version 10.2.5.0^86^ and run individual-based models with 500 iterations to account for demographic, environmental and genetic stochasticity. All values in VORTEX were kept at their default setting except those indicated below. To evaluate long-term metapopulation persistence we projected the model to 50 years, although we evaluated the species status after the first 20 years to fit IUCN criteria. Individual-based models rely on independent outcomes of the fates of individuals, so they include stochasticity in demography (random variation in births and deaths), environment (variation in disease, predation, food availability, weather, natural catastrophes) and genetics (decrease of fitness due to inbreeding depression and loss of diversity due to random genetic drift^87^). All PVA models were carried out at the subpopulation level (n = 100) using the above mentioned current Spanish metapopulation structure^49^ (Fig. 5). We defined the species extinction as the absence of at least one sex.

We included inbreeding depression to introduce evolutionary processes in the model. Here, we use the default value of 6.29 lethal equivalents suggested by Lacy and Pollak^86^, which represents the combined effect of inbreeding on fecundity and first year survival reported by O’Grady et al.^88^. The correlation in the environmental variability among populations was fixed at an intermediate value of 50% (see also^81^). The value of the correlation between breeding and survival was also maintained at the default value of 50%^86^. Similar to Suárez and Carriles^81^, we included one regional catastrophe with a frequency of 5% of the years in which 5% of females would not breed and survival would suffer a reduction of 5%.

We built a base model considering the most plausible value of each population parameter regarding the available current information (see below and Table 2) and estimated the probability of metapopulation extinction after 20 years. Then, we ran alternative models to assess the impact on the species of critical parameters and potentially handled through management plans and habitat restoration: productivity; breeding females, survival of adults and juveniles, and survival during dispersion (see below). For each of these sensitivity models we varied the specified parameter and kept the rest constant, to avoid interactions and obtain comparative results, and all the simulations were run with 500 iterations. We used the variation range that provided a response from 0 to 100% in the probability of metapopulation extinction in 20 years. For each model we estimated the mean year growth rate (*r*), mean population size each 10-year period (*N*_*t*_), probability of extinction each 10-year period (*P*_*0*_*(t)*: equivalent to the proportion of the 500 iterations in which the population is extinct or remains extant), median time to extinction (*T*_*med*_), mean time to extinction (*T*_*mean*_) and maximum time to extinction (*T*_*max*_, equivalent to the year in which all 500 iterations result in metapopulation extinction).

### Demographic parameters

Regarding dispersal, Dupont’s Lark adults appear to be resident^85,87–92^ while juveniles are assumed to be the dispersive fraction of the metapopulation^49,68^. Our base model assumed that only first year individuals of both sexes disperse and included 50% of dispersal survival. To avoid the use of a fixed arbitrary dispersal rate, we used the probability of connection between paired subpopulations considering juvenile movements of 20 km^49^ and a proportion of 10% of dispersers^79^.

In relation to longevity, our capture-recapture work during 2000–2018 in the Iberian Range showed a maximum lifespan of five years, despite some rare events of longer longevity (one 8-year-old bird recaptured^85^; 15 years used in Laiolo et al*.*^79^). Despite recent evidence of extra-pair copulation in this^82^ and other alaudids^93^, monogamy is considered the reproductive system in the Dupont’s Lark^77–81^. The age of reproduction was considered from year 1 to 5, concordant with the lifespan. Number of clutches per year was three, with a maximum of 5 eggs per clutch^82^. Sex ratio at birth was 50%.

As adult sex ratio is strongly male biased (0.79 following Vögeli et al*.*^51^; 0.61 following Suárez et al*.*^52^), the base model considered that all females were available to breed^85^. We introduced a random 10% SD in percent of breeding females to account for environmental variation. In this way, the value varied in each of the 500 iterations of the model within that range. We considered 80% of breeding males, due to the 20% of floating males registered by radiotracking^81^. We used distribution of broods as in previous works^79,81^, which estimated this parameter using data available on the Skylark^94^, in which 6, 29 and 65% of females lay 1, 2 and 3 clutches, respectively. Productivity of 1.5 ± 0.5 offspring/brood was based on the most recent study available^82^.

Regarding mortality rates we averaged the two data available, mean male mortality used in Laiolo et al*.*^79^ and in Vögeli et al*.*^90^ (54% and 50% respectively; mean = 52%). We estimated adult female mortality at 69% using the same method as Suárez and Carriles^81^. We considered that juveniles of both sexes suffer higher mortality rates than adults, and accordingly used the highest of both values available (69%). To account for stochasticity, as well as for percentage of breeding females, we introduced a variability of 10% SD in mortality of adults and juveniles.

Regarding initial population size, we used the most recent census data for each of the 100 subpopulations. We inferred the current number of males in 2020 applying the annual change rate of − 3.9%^36^. We then applied the sex ratio of 0.61^85^ to obtain the number of females and total population size per subpopulation in 2020 (see initial population size per subpopulation in Supplementary Table S1).

The carrying capacity (K) of each subpopulation was estimated based on the mean density of 1 individual/10 ha obtained by radiotracking^43^. To calculate the surface of adequate habitat within each subpopulation we first intersected the 16,676 observations from 2000 to 2018 with all available CORINE Land Cover layers in this period (2000, 2006, 2012 and 2018), and assigned to each point the most updated land category according to the observation date. We selected as the preferred categories of the species those accounting for 95% of the observations (Supplementary Table S8). We then extracted those categories from the last CORINE layer (2018) to obtain the current distribution of the species’ habitat and clipped them with the 100 subpopulations. This method implies a risk of surface overestimation, as it can include CORINE categories with little probability of occupation by the species (low % of total observations, see Supplementary Table S8), but with high surface within the subpopulation. To control for this, we applied a correction weighting the obtained surface by its habitat quality (mean probability of presence of the 1 × 1 km cells included in it^48^), reducing carrying capacity estimates in subpopulations with a large occupation of such land categories with low probability of presence. Finally, we discarded the surface with a slope over 15%, which is strongly avoided by the Dupont’s Lark^39–47^ and those habitat patches smaller than 20 ha, matching the minimum area requirements of the species^91^. For a similar procedure, see García-Antón et al*.*^48^. We included a 10% SD in K to account for environmental stochasticity. Finally, we considered the current habitat loss described recently for the Spanish metapopulation, which is considered one of the main threats for the species, and introduced in the base model an annual change rate of -3.9% in K^36^.

### Sensitivity analysis

We varied the following parameters to evaluate their effect on the metapopulation probability of extinction. For each of them, we used the range of values that allowed an observation of a 0–100% response in the probability of metapopulation extinction in 20 years: productivity (variation range of 1.2–1.9 offspring/brood by increases of 0.1), percentage of breeding females (range of 75–100%, increases of 5%), adult male survival (range of 30–100%, increases of 10%), adult female survival (range of 20–60%, increases of 10%), juvenile survival (range of 25–40%, increases of 2.5%) and dispersal survival (range of 0–100%, increases of 10%). See Supplementary Table S3.

### Critical areas

We used the base model to identify subpopulations of a higher concern and in need of special attention, following two criteria: imminent extinction and fast population decline. In the first case, we considered critical those subpopulations reaching extinction in the first 2 years (*T*_*mean*_ < 2 across the 500 iterations). Regarding population growth, we selected the subpopulations with a fastest annual decline (higher negative mean *r* values). We used a Pearson’s correlation test to evaluate the association between the mean time to extinction of each subpopulation and its initial population size and distance to the metapopulation centroid.

### Habitat restoration

We tested for the effects that a habitat management program applied in a subset of key subpopulations would have on metapopulation persistence. Assuming that the program would increase habitat suitability (more available foraging areas and/or safer nesting sites), we increased carrying capacity and productivity to simulate successful habitat restoration. We first generated nine different scenarios to evaluate the effect of spatial (number of subpopulations managed) and temporal (number of years of application) scale of the program on the overall metapopulation parameters. The selected subpopulations were those with a higher initial population size, and the nine scenarios tested were: (i) the 5 largest subpopulations during 3 consecutive years; (ii) 5 subpopulations/5 years; (iii) 5 subpopulations/10 years; (iv) 10 subpopulations/3 years; (v) 10 subpopulations/5 years; (vi) 10 subpopulations/10 years; (vii) 15 subpopulations/3 years; (viii) 15 subpopulations/5 years; and (ix) 15 subpopulations/10 years. We used two-way ANOVA test to evaluate whether the number of subpopulations and years of management had a significant effect on the metapopulation persistence, using mean time to extinction as the response variable and number of subpopulations and years as factors. Tukey's Honest Significant Difference test was employed to find differences among groups.

Based on the scenario that we considered most reliably applicable (10 subpopulations managed for 3 years), we tested the effect of increasing the intensity of the restoration on the selected subset of subpopulations. We assessed five different alternatives: 1, 5, 10, 15 and 20% increases in both productivity and carrying capacity. We evaluated the effects with one-way ANOVA and a Tukey’s post-hoc test.

### Translocation

We simulated the effects of a translocation program on the metapopulation viability, as well as on donor and recipient subpopulations. We selected donor subpopulations as those with more than 100 males (n = 7, Supplementary Table S6), all of them in the core area of the metapopulation. As recipients, we selected those seven subpopulations (same number as donors) with the most unfavorable situation based on the PVA results (shortest mean time to extinction). We simulated two different scenarios: recipients close to and distant from the metapopulation core (Supplementary Table S6).

We used a realistic time period of translocating individuals during three consecutive years. For each scenario, we simulated different harvesting alternatives varying the number of individuals moved from donor to recipient subpopulations yearly: 1 + 1, 2 + 2, 3 + 3, 5 + 5, 10 + 10 and 10 + 6 males/females, this last one adjusted to the species’ sex ratio. Movements were directed from donor to recipient subpopulations and individuals were randomized to increase allele diversity.

We carried out an ANOVA test to evaluate differences in the mean time to extinction of the metapopulation before and after the translocation, for the two different scenarios. We used harvesting alternatives (6 classes + no translocation) and close/distant scenario as factors. The response variable was the mean time to extinction obtained in the simulation. We tested differences in donor and recipient subpopulations using harvesting alternatives and subpopulation ID as factors, and *T*_*mean*_ as a response variable.

### Factors affecting population viability

We assessed the effect of geographic and climatic variables on the mean time to extinction (*T*_*mean*_) of the 100 subpopulations using a GLM. As geographic predictors, we included the linear distances to the nearest subpopulation (border to border) and to the metapopulation centroid (mean X and Y values of the centroids of all subpopulations). As climate predictors we included several variables obtained from the WorldClim database^95^ with 1 km resolution from the time series 2000–2018, concordant with our data set: minimum January temperature (to account for the coldest temperature of the year), maximum August temperature (as the hottest peak) and annual precipitation (cumulative rainfall of the 12 months). We obtained the mean value of the raster cells that intersected with the habitat patches within each subpopulation. For each of the three climate variables we calculated the overall average value and the 18-year trend, estimated as the slope of the linear regression for the whole time period. As the response variable, we used the value of mean time to extinction of the 100 subpopulations under the base model obtained in VORTEX. We used a Gaussian link, and model fit was assessed via examination of residuals. Starting from the saturated model and using the drop1 function in R Chambers^19^ we removed those variables that showed the highest p-value, subsequently running a new reduced model and comparing it to the previous one using a Log-Likelihood ratio Chi-squared test for the significance of the model. This procedure was carried out until all remaining variables showed a significant effect, and a significant difference between consecutive models tested using ANOVA was found. To avoid circularity, model parameters previously used to build the PVA, and therefore not independent of mean time to extinction, were not included in the GLM (such as initial population size, habitat surface and habitat quality).

## Supplementary Information

Supplementary Video 1.

Supplementary Information 1.

## Data Availability

All data generated or analyzed during this study are included in this published article (and its Supplementary Information files).

## References

[1] Powers RP, Jetz W (2019). Global habitat loss and extinction risk of terrestrial vertebrates under future land-use-change scenarios. Nat. Clim. Chang..

[2] Pardini R, Nichols E, Püttker T (2017). Biodiversity response to habitat loss and fragmentation. Encycl. Anthropocene.

[3] Fahrig L, Baudry J, Brotons L, Burel FG, Crist TO, Fuller RJ, Sirami C, Siriwardena GM, Martin JL (2011). Functional landscape heterogeneity and animal biodiversity in agricultural landscapes. Ecol. Lett..

[4] Moilanen A, Hanski I (1998). Metapopulation dynamics: Effects of hábitat quality and landscape structure. Ecology.

[5] Fahrig L (2003). Effects of habitat fragmentation on biodiversity. Annu. Rev. Ecol. Evol. Syst..

[6] Cornelius C, Awade M, Candia-Gallardo C, Sieving KE, Metzger JP (2017). Habitat fragmentation drives inter-population variation in dispersal behavior in a Neotropical rainforest bird. Perspect. Ecol. Conserv..

[7] Xu Y, Si Y, Wang Y, Zhang Y, Prins HHT, Cao L, de Boer WF (2019). Loss of functional connectivity in migration networks induces population decline in migratory birds. Ecol. Appl..

[8] Hens H, Pakanen V, Jäkäläniemi A, Tuomi JT, Kvist L (2017). Low population viability in small endangered orchid populations: Genetic variation, seedling recruitment and stochasticity. Biol. Cons..

[9] Silva JP, Correia R, Alonso H, Martins RC, D’Amico M, Delgado A, Sampaio H, Godinho C, Moreira F (2018). EU protected area network did not prevent a country wide population decline in a threatened grassland bird. PeerJ.

[10] Gaget E, Fay R, Audiron S, Villers A, Bretagnolle V (2019). Long-term decline despite conservation efforts questions Eurasian Stone-curlew population viability in intensive farmlands. Ibis.

[11] van Oosten HH, van den Burg AB, Arlt D, Both C, van den Brink NW, Chiu S, Crump D, Jeppsson T, de Kroon H, Traag W, Siepel H (2019). Hatching failure and accumulation of organic pollutants through the terrestrial food web of a declining songbird in Western Europe. Sci. Total Environ..

[12] Brambilla M, Gustin M, Vitulano S, Falco R, Bergero V, Negri I, Bogliani G, Celada C (2017). Sixty years of habitat decline: impact of land-cover changes in northern Italy on the decreasing ortolan bunting Emberiza hortulana. Reg. Environ. Change.

[13] Heldbjerg H, Sunde P, Fox AD (2018). Continuous Population Declines for Specialist Farmland Birds 1987–2014 in Denmark Indicates No Halt in Biodiversity Loss in Agricultural Habitats.

[14] Traba J, Morales MB (2019). The decline of farmland birds in Spain is strongly associated with the loss of fallowland. Sci. Rep..

[15] Reif J, Vermouzek Z (2018). Collapse of farmland bird populations in an Eastern European country following its EU accession. Conserv. Lett..

[16] Levins, R. Extinction. In: Some mathematical problems in biology. Mathematical Society of America, Providence, R.I. Pages 77–107 (1970).

[17] Hanski I (1999). Metapopulation ecology.

[18] Johnson MD (2007). Measuring habitat quality: A review. Condor.

[19] Vögeli M, Serrano D, Pacios F, Tella JL (2010). The relative importance of patch habitat quality and landscape attributes on a declining steppe-bird metapopulation. Biol. Cons..

[20] Traba J, Sastre P, Morales MB, Morales MB, Traba J (2013). Factors determining species richness and composition of steppe bird communities in peninsular Spain: grass-steppe vs. shrub-steppe bird species. Steppe Ecosystems.

[21] Burfield IJ, Bota G, Morales MB, Mañosa S, Camprodon J (2005). The conservation status of steppic birds in Europe. Ecology and Conservation of Steppe-Land Birds.

[22] Donald PF, Sanderson FJ, Burfield IJ, van Bommel FPJ (2006). Further evidence of continent-wide impacts of agricultural intensification on European farmland birds, 1990–2000. Agr. Ecosyst. Environ..

[23] Burfield I, van Bommel F (2004). Birds in Europe: Population Estimates, Trends and Conservation Status.

[24] Benton TG, Vickery JA, Wilson JD (2003). Farmland biodiversity: is habitat heterogeneity the key?. Trends Ecol. Evol..

[25] Santos T, Suárez F, Bota G, Morales MB, Mañosa S, Camprodon J (2005). Biogeography and population trends of iberian steppe bird. Ecology and Conservation of Steppe-Land Birds.

[26] Gómez-Catasús J, Garza V, Traba J (2018). Wind farms affect the occurrence, abundance and population trends of small passerine birds: The case of the Dupont’s Lark. J. Appl. Ecol..

[27] Donald PF, Green R, Heath MF (2001). Agricultural intensification and the collapse of Europe's farmland bird populations. Proc. R. Soc. Ser. B..

[28] Brotons L, Mañosa S, Estrada J (2004). Modelling the effects of irrigation schemes on the distribution of steppe birds in Mediterranean farmland. Biodivers. Conserv..

[29] Madroño A, González C, Atienza JC (2004). Libro rojo de las aves de España.

[30] Concepción ED, Díaz M (2013). Medidas agroambientales y conservación de la biodiversidad: Limitaciones y perspectivas de futuro. Ecosistemas.

[31] Traba J (2020). Intensificación agrícola y efectos sobre las aves. Revista de la Sociedad Cordobesa de Historia Natural.

[32] Prévosto B, Kuiters L, Bernhardt-Römermann M (2011). Impacts of land abandonment on vegetation: Successional pathways in European habitats. Folia Geobot.

[33] García-Tejero S, Taboada A, Tárrega R, Salgado JM (2013). Land use changes and ground dwelling beetle conservation in extensive grazing dehesa systems of north-west Spain. Biol. Cons..

[34] Dennis P, Skartveit J, McCracken D, Pakeman R, Beaton K, Kunaver A, Evans D (2008). The effects of livestock grazing on foliar arthropods associated with bird diet in upland grasslands of Scotland. J. Appl. Ecol..

[35] BirdLife International (2015). European Red List of Birds.

[36] Gómez-Catasús J, Pérez-Granados C, Barrero A, Bota G, Giralt D, López-Iborra GM, Serrano D, Traba J (2018). European population trends and current conservation status of an endangered steppe-bird species: the Dupont’s Lark Chersophilus duponti. PeerJ.

[37] de Juana E, Suárez F, del Hoyo J, Elliott A, Sargatal J, Christie DA, de Juana E (2020). Dupont’s Lark (Chersophilus duponti), version 1.0. Birds of the World.

[38] García JT, Suárez F, Garza V, Calero-Riestra M, Hernández J, Pérez-Tris J (2008). Genetic and phenotypic variation among geographically isolated populations of the globally threatened Dupont’s Lark Chersophilus duponti. Mol. Phylogenet. Evol..

[39] Méndez M, Tella JL, Godoy JA (2011). Restricted gene flow and genetic drift in recently fragmented populations of an endangered steppe bird. Biol. Cons..

[40] Méndez M, Vögeli M, Tella JL, Godoy JA (2014). Joint effects of population size and isolation on genetic erosion in fragmented populations: Finding fragmentation thresholds for management. Evol. Appl..

[41] Garza V, Suárez F (1990). Distribución, población y selección de hábitat de la Alondra de Dupont (Chersophilus duponti) en la Península Ibérica. Ardeola.

[42] Martín-Vivaldi M, Marín JM, Archila F, López E, De Manuel LC (1999). Caracterización de una nueva población reproductora de Alondra de Dupont (*Chersophilus duponti*) (Passeriformes, Alaudidae) en el Sureste Ibérico. Zool. Baetica.

[43] Garza V, Suárez F, Herranz J, Traba J, De la Morena ELG, Morales MB, González R, Castañeda M (2005). Home range, territoriality and habitat selection by the Dupont’s Lark Chersophilus duponti during the breeding and postbreeding periods. Ardeola.

[44] Seoane J, Justribó JH, García F, Retamar J, Rabadan C, Atienza JC (2006). Habitat-suitability modelling to assess the effects of land-use changes on Dupont’s Lark *Chersophilus duponti*: A case study in the Layna Important Bird Area. Biol. Cons..

[45] Nogués-Bravo D, Agirre A (2006). Patrón y modelos de distribución espacial de la alondra ricotí Chersophilus duponti durante el periodo reproductor en el LIC de Ablitas (Navarra). Ardeola.

[46] García JT, Suárez F, Garza V, Justribó JH, Oñate JJ, Hervás I, Calero M, Morena EL (2008). Assessing the distribution, habitat, and population size of the threatened Dupont’s Lark Chersophilus duponti in Morocco: Lessons for conservation. Oryx.

[47] Pérez-Granados C, López-Iborra GM, Seoane J (2017). A multi-scale analysis of habitat selection in peripheral populations of the endangered Dupont’s Lark Chersophilus duponti. Bird Conserv. Int..

[48] García-Antón A, Garza V, Hernández-Justribó J, Traba J (2019). Factors affecting Dupont’s Lark distribution and range regression in Spain. PLoS ONE.

[49] García-Antón, A., Garza, V. & Traba, J. (in press). Connectivity in Spanish metapopulation of Dupont’s Lark may be maintained by dispersal over medium 3 distances and stepping stones. *PeerJ*. 10.7717/peerj.11925 PMC8380426 34466286

[50] Traba, J., Garza, V., García-Antón, A., Gómez-Catasús, J., Zurdo, J., Pérez-Granados, C., Morales, M. B., Oñate, J. J., Herranz, J., Malo, J. Criterios para la gestión y conservación de la población española de alondra ricotí Chersophilus duponti. Fundación Biodiversidad, Ministerio de Agricultura, Alimentación y Medio Ambiente. Madrid. (2019).

[51] Vögeli M, Serrano D, Tella JL, Méndez M, Godoy JA (2007). Sex determination of Dupont´s lark Chersophilus duponti using molecular sexing and discriminant functions. Ardeola.

[52] Suárez F, García JT, Carriles E, Calero M, Agirre A, Justribó JH, Garza V (2009). Sex-ratios of an endangered lark after controlling for a male-biased sampling. Ardeola.

[53] Garza, V., Suárez, F., Tella, J. L. Alondra de Dupont, Chersophilus duponti. In: Madroño, A., González, C., Atienza, J. C. (eds). Libro Rojo de las Aves de España. Madrid: Dirección General para la Biodiversidad-SEO/BirdLife pp 309–312. (2004).

[54] Íñigo, A., Garza, V., Tella, J. L., Laiolo, P., Suárez, F., Barov, B. Action Plan for the Dupont’s Lark Chersophilus duponti in the European Union. SEO/Birdlife – BirdLife International –Comisión Europea. (2008).

[55] Gómez-Catasús J, Garza V, Morales MB (2019). Hierarchical habitat-use by an endangered steppe bird in fragmented landscapes is associated with large connected patches and high food availability. Sci Rep.

[56] Reverter M, Gómez-Catasús J, Barrero A, Pérez-Granados C, Bustillo D, Traba J (2019). Interactions in shrub-steppes: Implications for the maintenance of a threatened bird. Ecosistemas.

[57] Serrano D, Margalida A, Pérez-García JM, Juste J, Traba J, Varela F, Carrete M, Aihartza J, Real J, Mañosa S, Flaquer C, Marin I, Morales MB, Alcalde JT, Arroyo B, Sánchez-Zapata JA, Blanco G, Negro JJ, Tella JL, Ibáñez C, Tellería JL, Hiraldo F, Donázar JA (2020). Renewables in Spain threaten biodiversity. Science.

[58] Pe'er G, Zinngrebe Y, Moreira F, Sirami C, Schindler S, Müller R, Lakner S (2019). A greener path for the EU common agricultural policy. Science.

[59] Bland, L. M., Keith, D. A., Miller, R. M., Murray, N. J. & Rodríguez, J. P. (eds.) Guidelines for the application of IUCN Red List of Ecosystems Categories and Criteria, Version 1.1. Gland, Switzerland: IUCN. ix + 99pp. (2017).

[60] Flather CH, Hayward GD, Beissinger SR, Stephens PA (2011). Minimum viable populations: Is there a 'magic number' for conservation practitioners?. Trends Ecol. Evol..

[61] Carvajal MA, Alaniz AJ, Smith-Ramírez C, Sieving KE (2018). Assessing habitat loss and fragmentation and their effects on population viability of forest specialist birds: Linking biogeographical and population approaches. Divers. Distrib..

[62] Trask AE, Fenn S, Bignal EM, McCracken DI, Monaghan P, Reid JM (2019). Evaluating the efficacy of independent versus simultaneous management strategies to address ecological and genetic threats to population viability. J. Appl. Ecol..

[63] Akçakaya HR, Sjögren-Gulve P (2000). Population viability analyses in conservation planning: An overview. Ecol. Bull..

[64] Frankham R, Ballou J, Briscoe D, McInnes K (2002). Introduction to Conservation Genetics.

[65] Pulliam HR (1988). Sources, sinks, and population regulation. Am. Nat..

[66] Bota G, Giralt D, Guixé DL (2016). Alondra Ricotí en Cataluña: evolución histórica de una población en el límite del área de distribución.

[67] Pérez-Granados C, Bota G, Giralt D, Traba J (2018). A cost-effective protocol for monitoring birds using autonomous recording units: A case study with a night-time singing passerine. Bird Study.

[68] García-Antón, A., Garza, V. & Traba, J. Dispersión de más de 30 km en un macho de primer año de alondra ricotí (Chersophilus duponti) en el Sistema Ibérico. I Workshop Nacional de la Alondra ricotí Chersophilus duponti: Estrategias Futuras. Estación Ornitológica de Padul, Granada. 13 junio (2015).

[69] Kauhala K, Helle P, Helle E (2000). Predator control and the density and reproductive success of grouse populations in Finland. Ecography.

[70] Fletcher K, Aebischer NJ, Baines D, Foster R, Hoodless AN (2010). Changes in breeding success and abundance of ground-nesting moorland birds in relation to the experimental deployment of legal predator control. J. Appl. Ecol..

[71] Banks PB, Dickman CR, Newsome AE (1998). Ecological costs of feral predator control: Foxes and rabbits. J. Wildl. Manag..

[72] Bolton M, Tyler G, Smith K, Bamford R (2007). The impact of predator control on lapwing Vanellus vanellus breeding success on wet grassland nature reserves. J. Appl. Ecol..

[73] Walsh JC, Wilson KA, Benshemesh J, Possingham HP (2012). Unexpected outcomes of invasive predator control: The importance of evaluating conservation management actions. Anim. Conserv..

[74] Oro D, Margalida A, Carrete M, Heredia R, Donázar JA (2008). Testing the goodness of supplementary feeding to enhance population viability in an endangered vulture. PLoS ONE.

[75] Ruffino L, Salo P, Koivisto E (2014). Reproductive responses of birds to experimental food supplementation: A meta-analysis. Front. Zool..

[76] Cuesta D, Taboada A, Calvo L, Salgado JM (2008). Short- and medium-term effects of experimental nitrogen fertilization on arthropods associated with Calluna vulgaris heathlands in north-west Spain. Environ. Pollut..

[77] Estrada A, Delgadom MP, Arroyo B, Traba J, Morales MB (2016). Forecasting large-scale habitat suitability of European bustards under climate change: The role of environmental and geographic variables. PLoS ONE.

[78] Zhang X, Johnston ER, Li L, Konstantinidis KT, Han X (2017). Experimental warming reveals positive feedbacks to climate change in the Eurasian Steppe. ISME J..

[79] Laiolo P, Vögeli M, Serrano D, Tella JL (2008). Song diversity predicts the viability of fragmented bird populations. PLoS ONE.

[80] Traba, J., García de la Morena, E. L. & Garza, V. Análisis de Viabilidad de Poblaciones como herramienta para el diseño de Parques Eólicos. El caso de las poblaciones de alondra ricotí (Chersophilus duponti) del sur de Soria. I Congreso Ibérico sobre Energía Eólica y Conservación de Fauna. Jerez de la Frontera, Cádiz (2011).

[81] Suárez, F. & Carriles, E. Análisis de viabilidad poblacional. In: Suárez, F. (ed.) *La alondra ricotí* (Chersophilus duponti), pp. 319–326. Dirección General para la Biodiversidad. Ministerio de Medio Ambiente y Medio Rural y Marino. Madrid (2010).

[82] Pérez-Granados C, López-Iborra GM, Garza V, Traba J (2017). Breeding biology of the endangered Dupont’s Lark *Chersophilus duponti* in two separate Spanish shrub-steppes. Bird Study.

[83] Pérez-Granados C, López-Iborra GM (2014). ¿Por qué la alondra ricotí debe catalogarse como “En peligro de Extinción”?. Quercus.

[84] Pérez-Granados C, López-Iborra GM (2013). Census of breeding birds and population trends of the Dupont’s Lark Chersophilus duponti in eastern Spain. Ardeola.

[85] Suárez, F. *La alondra ricotí (Chersophilus duponti)*. Dirección General para la Biodiversidad. Ministerio de Medio Ambiente y Medio Rural y Marino Medio Rural y Marino, Madrid. 525 pp (2010).

[86] Lacy, R. C., & Pollak, J. P. Vortex: A Stochastic Simulation of the Extinction Process. Version 10.2.9. Chicago Zoological Society, Brookfield, Illinois, USA (2017).

[87] Lacy RC (2000). Considering Threats to the Viability of Small Populations Using Individual-Based Models. Ecol. Bull..

[88] Ogrady JJ, Brook BW, Reed DH, Ballou JD, Tonkyn DW, Richard Frankham R (2006). Realistic levels of inbreeding depression strongly affect extinction risk in wild populations. Biol. Conserv..

[89] Laiolo P, Vögeli M, Serrano D, Tella JL (2007). Testing acoustic versus physical marking: Two complementary methods for individual-based monitoring of elusive species. J. Avian Biol..

[90] Vögeli M, Laiolo P, Serrano D, Tella JL (2008). Who are we sampling? Apparent survival differs between methods in a secretive species. Oikos.

[91] Chambers, J. M. (1992) *Linear models.* Chapter 4 of *Statistical Models in *eds J. M. Chambers and T. J. Hastie, Wadsworth & Brooks/Cole.

[92] Pérez-Granados, C. & López-Iborra, G. M. Baja dispersión adulta y baja tasa de recaptura juvenil de la alondra ricotí (Chersophilus duponti) en el Rincón de Ademuz (Valencia). XX Iberian Ringing Congress (2015).

[93] Briefer E, Rybak F, Aubin T (2008). When to be a dear enemy: flexible acoustic relationships of neighbouring skylarks *Alauda arvensis*. Anim. Behav..

[94] Delius JD (1965). A population study of Skylarks Alauda arvensis. Ibis.

[95] Hijmans RJ, Cameron SE, Parra JL, Jones PG, Jarvis A (2005). Very high resolution interpolated climate surfaces for global land areas. Int. J. Climatol..

