# Supplementary material for: Population viability analysis of the endangered Dupont’s Lark Chersophilus duponti in Spain
Source: Sci Rep. 2021 Oct 7;11:19947. doi: 10.1038/s41598-021-99125-y (PMC8497488; doi:10.1038/s41598-021-99125-y)
Supplement: Supplementary file 2 — Supplementary Information 1. [file 41598_2021_99125_MOESM2_ESM.docx]

**Population viability analysis of the endangered Dupont’s Lark *Chersophilus duponti* in Spain**

Alexander García-Antón^1^. Juan Traba^1.2*^

^1^ Terrestrial Ecology Group (TEG-UAM). Department of Ecology. Universidad Autónoma de Madrid. C/ Darwin. 2. 28049. Madrid. Spain.

^2^ Centro de Investigación en Biodiversidad y Cambio Global. Universidad Autónoma de Madrid (CIBC-UAM). C/ Darwin. 2. 28049. Madrid. Spain.

*Corresponding author

Email: [juan.traba@uam.es](mailto:juan.traba@uam.es)

**Supplementary File S1.** Graphic animation of the extinction process of Dupont’s Lark in Spain, representing the results of the base model and showing extinctions, recolonizations and range contraction (represented by the MCP of the extant subpopulations). The colour of extant subpopulations indicates probability of extinction from 0% (yellow) to 1% (red).

**Supplementary Table S1.** Dupont’s Lark PVA base model results for the 100 subpopulations and the metapopulation. Estimated initial population size in 2020 (*N_0_*); mean population growth rate (*r*) with standard deviation (*SD_r_*) and 95% confidence interval (*CI_r_*) across 500 iterations; mean population size in *t* years (*N_t_*); probability of extinction in *t* years (*P_0_(t)*: equivalent to the proportion of the 500 iterations in which the population is extinct or remains extant); *T_med_*: median time to extinction of the 500 iterations; *T_mean_*: mean time to extinction of the 500 iterations; *T_max_*: maximum time to extinction (equivalent to the year in which all 500 iterations result in metapopulation is extinction); *Dist. N*: linear distance to the closest neighbour subpopulation (border to border); *Dist. C*: linear distance to the metapopulation centroid.

| **ID** | **Subpopulation** | **Prov.** | ***N_0_*** | ***r*** | ***SD(r)*** | ***CI(r)*** | ***N_10_*** | ***SD(N_10_)*** | ***CI(N_10_)*** | ***P_0_(10)*** | ***N_20_*** | ***SD(N_20_)*** | ***CI(N_20_)*** | ***P_0_(20)*** | ***T_med_*** | ***T_mean_*** | ***SD(T_mean_)*** | ***CI(T_mean_)*** | ***T_max_*** | ***Dist. N*** | ***Dist. C*** |
| --- | --- | --- | --- | --- | --- | --- | --- | --- | --- | --- | --- | --- | --- | --- | --- | --- | --- | --- | --- | --- | --- |
| 1 | Fariza | ZA | 9 | -0.194 | 0.395 | 0.035 | 0.000 | 0.000 | 0.000 | 1.000 | 0.000 | 0.000 | 0.000 | 1.000 | 3 | 3.32 | 1.54 | 0.13 | 9 | 15.3 | 362.1 |
| 2 | Donadillo | ZA | 1 | 0.000 | 0.000 | 0.000 | 0.000 | 0.000 | 0.000 | 1.000 | 0.000 | 0.000 | 0.000 | 1.000 | 1 | 1.00 | 0.00 | 0.00 | 1 | 14.0 | 382.0 |
| 3 | Villanueva de Valrojo | ZA | 1 | 0.000 | 0.000 | 0.000 | 0.000 | 0.000 | 0.000 | 1.000 | 0.000 | 0.000 | 0.000 | 1.000 | 1 | 1.00 | 0.04 | 0.00 | 3 | 14.0 | 368.3 |
| 4 | Tábara | ZA | 11 | -0.268 | 0.382 | 0.034 | 2.000 | 0.000 | 0.000 | 0.998 | 0.000 | 0.000 | 0.000 | 1.000 | 4 | 4.39 | 1.82 | 0.16 | 11 | 9.5 | 347.2 |
| 5 | Vegalatrave | ZA | 1 | 0.269 | 0.367 | 0.032 | 0.000 | 0.000 | 0.000 | 1.000 | 0.000 | 0.000 | 0.000 | 1.000 | 1 | 1.04 | 0.28 | 0.02 | 7 | 9.5 | 350.7 |
| 6 | Villaseco del Pan | ZA | 17 | -0.281 | 0.369 | 0.032 | 3.530 | 1.130 | 0.099 | 0.970 | 0.000 | 0.000 | 0.000 | 1.000 | 5 | 5.64 | 2.18 | 0.19 | 13 | 3.4 | 339.5 |
| 7 | Almaraz de Duero | ZA | 18 | -0.289 | 0.372 | 0.033 | 4.650 | 3.690 | 0.323 | 0.960 | 0.000 | 0.000 | 0.000 | 1.000 | 6 | 5.85 | 2.22 | 0.19 | 14 | 3.4 | 327.7 |
| 8 | Velilla de la Sierra | SO | 1 | 0.190 | 0.323 | 0.028 | 0.000 | 0.000 | 0.000 | 1.000 | 0.000 | 0.000 | 0.000 | 1.000 | 1 | 1.07 | 0.37 | 0.03 | 6 | 3.7 | 106.0 |
| 9 | Arancón | SO | 2 | 0.175 | 0.384 | 0.034 | 0.000 | 0.000 | 0.000 | 1.000 | 0.000 | 0.000 | 0.000 | 1.000 | 1 | 1.69 | 1.04 | 0.09 | 6 | 2.4 | 100.8 |
| 10 | Aldealpozo | SO | 4 | -0.134 | 0.380 | 0.033 | 0.000 | 0.000 | 0.000 | 1.000 | 0.000 | 0.000 | 0.000 | 1.000 | 2 | 2.26 | 1.25 | 0.11 | 8 | 2.4 | 97.5 |
| 11 | Pozalmuro | SO | 1 | 0.118 | 0.311 | 0.027 | 0.000 | 0.000 | 0.000 | 1.000 | 0.000 | 0.000 | 0.000 | 1.000 | 1 | 1.09 | 0.45 | 0.04 | 7 | 0.1 | 96.4 |
| 12 | Pinilla del Campo | SO | 3 | -0.005 | 0.363 | 0.032 | 0.000 | 0.000 | 0.000 | 1.000 | 0.000 | 0.000 | 0.000 | 1.000 | 2 | 2.01 | 1.23 | 0.11 | 9 | 0.1 | 88.1 |
| 13 | Dévanos | SO | 4 | -0.099 | 0.413 | 0.036 | 2.000 | 0.000 | 0.000 | 0.998 | 0.000 | 0.000 | 0.000 | 1.000 | 2 | 2.52 | 1.53 | 0.13 | 12 | 14.0 | 110.2 |
| 14 | Aranda de Moncayo | Z | 36 | -0.301 | 0.342 | 0.030 | 6.150 | 5.200 | 0.456 | 0.830 | 0.000 | 0.000 | 0.000 | 1.000 | 8 | 7.78 | 2.65 | 0.23 | 18 | 5.3 | 70.3 |
| 15 | Deza | SO | 1 | 0.108 | 0.418 | 0.037 | 3.000 | 0.000 | 0.000 | 0.996 | 0.000 | 0.000 | 0.000 | 1.000 | 1 | 1.40 | 1.08 | 0.09 | 14 | 5.3 | 60.9 |
| 16 | Ablitas | NA | 28 | -0.268 | 0.347 | 0.030 | 5.540 | 3.290 | 0.288 | 0.864 | 0.000 | 0.000 | 0.000 | 1.000 | 7 | 7.68 | 2.61 | 0.23 | 18 | 17.1 | 119.5 |
| 17 | Bardenas | NA | 106 | -0.252 | 0.297 | 0.026 | 20.600 | 18.020 | 1.580 | 0.188 | 7.210 | 5.020 | 0.440 | 0.962 | 13 | 13.48 | 3.51 | 0.31 | 25 | 20.4 | 150.0 |
| 18 | Talamantes | Z | 1 | 0.038 | 0.363 | 0.032 | 2.000 | 0.000 | 0.000 | 0.998 | 0.000 | 0.000 | 0.000 | 1.000 | 1 | 1.05 | 0.40 | 0.04 | 11 | 17.1 | 97.8 |
| 19 | Tardienta | HU | 8 | -0.249 | 0.394 | 0.034 | 5.000 | 0.000 | 0.000 | 0.998 | 0.000 | 0.000 | 0.000 | 1.000 | 3 | 3.62 | 1.85 | 0.16 | 11 | 29.2 | 161.0 |
| 20 | Ballobar | HU | 17 | -0.273 | 0.360 | 0.032 | 4.670 | 2.870 | 0.252 | 0.970 | 0.000 | 0.000 | 0.000 | 1.000 | 6 | 5.85 | 2.12 | 0.19 | 13 | 20.2 | 190.6 |
| 21 | Alfés | L | 11 | -0.148 | 0.381 | 0.033 | 0.000 | 0.000 | 0.000 | 1.000 | 0.000 | 0.000 | 0.000 | 1.000 | 2 | 2.05 | 1.11 | 0.10 | 9 | 34.7 | 230.9 |
| 22 | Páramo de Corcos | BU | 14 | -0.210 | 0.384 | 0.034 | 3.920 | 2.080 | 0.182 | 0.904 | 0.000 | 0.000 | 0.000 | 1.000 | 6 | 6.25 | 2.59 | 0.23 | 16 | 8.1 | 166.5 |
| 23 | Hoces del Riaza | SG | 85 | -0.267 | 0.307 | 0.027 | 10.800 | 7.710 | 0.676 | 0.356 | 5.000 | 0.000 | 0.000 | 0.998 | 11 | 11.62 | 2.81 | 0.25 | 22 | 8.1 | 145.5 |
| 24 | Los Castros | SG | 6 | -0.001 | 0.464 | 0.041 | 4.250 | 2.580 | 0.226 | 0.840 | 0.000 | 0.000 | 0.000 | 1.000 | 5 | 5.45 | 3.33 | 0.29 | 16 | 1.0 | 154.9 |
| 25 | Hoces del Duratón | SG | 94 | -0.305 | 0.305 | 0.027 | 10.740 | 8.550 | 0.749 | 0.460 | 2.000 | 0.000 | 0.000 | 0.998 | 11 | 10.94 | 2.76 | 0.24 | 21 | 1.0 | 154.1 |
| 26 | Cenegro | SO | 3 | 0.032 | 0.735 | 0.064 | 12.880 | 9.500 | 0.833 | 0.210 | 3.000 | 1.150 | 0.101 | 0.992 | 12 | 12.71 | 3.00 | 0.26 | 23 | 1.4 | 128.2 |
| 27 | Atauta | SO | 5 | 0.069 | 0.638 | 0.056 | 7.020 | 3.590 | 0.315 | 0.336 | 0.000 | 0.000 | 0.000 | 1.000 | 11 | 11.36 | 2.43 | 0.21 | 19 | 1.6 | 123.5 |
| 28 | Las Fraguas | SO | 9 | -0.219 | 0.405 | 0.036 | 3.380 | 1.600 | 0.140 | 0.984 | 0.000 | 0.000 | 0.000 | 1.000 | 4 | 4.47 | 2.07 | 0.18 | 14 | 5.4 | 106.0 |
| 29 | Nafría la Llana | SO | 8 | -0.162 | 0.396 | 0.035 | 3.430 | 1.160 | 0.102 | 0.972 | 0.000 | 0.000 | 0.000 | 1.000 | 5 | 4.88 | 2.33 | 0.20 | 15 | 0.7 | 103.9 |
| 30 | Fuentelárbol | SO | 1 | -0.017 | 0.450 | 0.039 | 4.290 | 2.140 | 0.188 | 0.918 | 0.000 | 0.000 | 0.000 | 1.000 | 4 | 4.76 | 3.14 | 0.28 | 16 | 0.7 | 100.3 |
| 31 | Brías | SO | 8 | -0.044 | 0.564 | 0.049 | 17.420 | 12.970 | 1.137 | 0.176 | 5.400 | 3.780 | 0.331 | 0.990 | 13 | 13.26 | 3.13 | 0.27 | 21 | 0.1 | 91.3 |
| 32 | Altos de Barahona | SO | 1125 | -0.448 | 0.263 | 0.023 | 17.680 | 13.380 | 1.173 | 0.086 | 4.000 | 0.000 | 0.000 | 0.998 | 13 | 13.27 | 2.17 | 0.19 | 21 | 0.1 | 71.6 |
| 33 | Hiendelaencina | GU | 12 | -0.118 | 0.396 | 0.035 | 5.970 | 3.840 | 0.337 | 0.682 | 0.000 | 0.000 | 0.000 | 1.000 | 9 | 9.11 | 2.86 | 0.25 | 19 | 10.5 | 82.8 |
| 34 | Conquezuela | SO | 4 | 0.057 | 0.717 | 0.063 | 9.350 | 5.470 | 0.479 | 0.248 | 2.000 | 0.000 | 0.000 | 0.998 | 12 | 12.00 | 2.61 | 0.23 | 22 | 0.2 | 57.5 |
| 35 | Sigüenza | GU | 88 | -0.228 | 0.315 | 0.028 | 16.400 | 11.520 | 1.010 | 0.138 | 4.330 | 2.310 | 0.202 | 0.994 | 13 | 13.34 | 2.78 | 0.24 | 24 | 3.2 | 58.0 |
| 36 | Layna | SO | 761 | -0.453 | 0.269 | 0.024 | 11.250 | 7.640 | 0.670 | 0.196 | 0.000 | 0.000 | 0.000 | 1.000 | 12 | 12.21 | 2.11 | 0.18 | 19 | 0.2 | 27.5 |
| 37 | La Torresaviñán | GU | 23 | -0.160 | 0.368 | 0.032 | 9.240 | 6.740 | 0.591 | 0.370 | 0.000 | 0.000 | 0.000 | 1.000 | 11 | 11.29 | 2.73 | 0.24 | 20 | 2.1 | 43.9 |
| 38 | Hortezuela de Océn | GU | 4 | 0.152 | 0.566 | 0.050 | 3.210 | 1.310 | 0.115 | 0.840 | 0.000 | 0.000 | 0.000 | 1.000 | 7 | 7.15 | 2.48 | 0.22 | 16 | 1.6 | 31.9 |
| 39 | Riba de Saelices | GU | 19 | -0.197 | 0.372 | 0.033 | 4.880 | 2.730 | 0.239 | 0.792 | 0.000 | 0.000 | 0.000 | 1.000 | 8 | 8.22 | 2.68 | 0.23 | 18 | 3.1 | 23.1 |
| 40 | Cueva de la Hoz | GU | 1 | 0.387 | 0.538 | 0.047 | 2.610 | 0.780 | 0.068 | 0.954 | 0.000 | 0.000 | 0.000 | 1.000 | 3 | 2.99 | 1.90 | 0.17 | 14 | 1.6 | 26.2 |
| 41 | Maranchón | GU | 1 | -0.147 | 0.390 | 0.034 | 7.920 | 5.950 | 0.522 | 0.428 | 0.000 | 0.000 | 0.000 | 1.000 | 11 | 10.85 | 2.63 | 0.23 | 19 | 1.8 | 17.3 |
| 42 | Alhama de Aragón | Z | 2 | 0.340 | 0.436 | 0.038 | 2.130 | 0.350 | 0.031 | 0.984 | 0.000 | 0.000 | 0.000 | 1.000 | 1 | 1.78 | 1.08 | 0.09 | 12 | 7.6 | 42.4 |
| 43 | Milmarcos-Llumes | GU | 50 | -0.239 | 0.319 | 0.028 | 8.160 | 5.490 | 0.481 | 0.414 | 0.000 | 0.000 | 0.000 | 1.000 | 11 | 10.97 | 2.69 | 0.24 | 20 | 0.1 | 21.7 |
| 44 | Paramera de Molina | GU | 233 | -0.350 | 0.291 | 0.025 | 11.700 | 8.800 | 0.771 | 0.198 | 2.000 | 0.000 | 0.000 | 0.998 | 12 | 12.33 | 2.51 | 0.22 | 21 | 0.1 | 10.2 |
| 45 | Torralba de los Frailes | TE | 16 | -0.131 | 0.372 | 0.033 | 7.880 | 5.550 | 0.486 | 0.462 | 6.000 | 0.000 | 0.000 | 0.996 | 10 | 10.60 | 3.15 | 0.28 | 24 | 0.5 | 33.5 |
| 46 | Calatayud | Z | 126 | -0.277 | 0.294 | 0.026 | 18.420 | 17.070 | 1.496 | 0.220 | 5.760 | 4.240 | 0.372 | 0.984 | 13 | 12.95 | 3.25 | 0.28 | 23 | 6.5 | 53.1 |
| 47 | Campo Romanos | Z | 21 | -0.209 | 0.364 | 0.032 | 5.750 | 4.190 | 0.367 | 0.722 | 0.000 | 0.000 | 0.000 | 1.000 | 8 | 8.20 | 8.20 | 0.72 | 19 | 6.5 | 57.8 |
| 48 | Segura de los Baños | TE | 226 | -0.297 | 0.283 | 0.025 | 23.810 | 20.900 | 1.832 | 0.098 | 7.360 | 5.360 | 0.470 | 0.972 | 14 | 14.03 | 2.97 | 0.26 | 27 | 5.5 | 85.0 |
| 49 | Lechago | TE | 2 | 0.127 | 0.538 | 0.047 | 3.940 | 1.850 | 0.162 | 0.896 | 0.000 | 0.000 | 0.000 | 1.000 | 4 | 4.45 | 3.12 | 0.27 | 19 | 0.2 | 61.2 |
| 50 | Cutanda | TE | 13 | -0.142 | 0.375 | 0.033 | 5.050 | 3.030 | 0.266 | 0.724 | 0.000 | 0.000 | 0.000 | 1.000 | 8 | 8.14 | 2.99 | 0.26 | 17 | 0.2 | 63.9 |
| 51 | Castilnovo | GU | 7 | -0.063 | 0.421 | 0.037 | 6.070 | 3.870 | 0.339 | 0.710 | 0.000 | 0.000 | 0.000 | 1.000 | 8 | 8.33 | 3.32 | 0.29 | 20 | 0.2 | 13.1 |
| 52 | Río Gallo | GU | 133 | -0.306 | 0.295 | 0.026 | 11.590 | 8.860 | 0.777 | 0.270 | 4.670 | 1.530 | 0.134 | 0.994 | 12 | 12.08 | 2.83 | 0.25 | 24 | 0.2 | 16.5 |
| 53 | Blancas | TE | 253 | -0.319 | 0.284 | 0.025 | 20.580 | 17.160 | 1.504 | 0.134 | 4.330 | 3.010 | 0.264 | 0.988 | 14 | 13.62 | 2.89 | 0.25 | 22 | 3.2 | 31.6 |
| 54 | Puerto Bañón | TE | 3 | 0.044 | 0.609 | 0.053 | 7.720 | 4.580 | 0.401 | 0.466 | 4.000 | 0.000 | 0.000 | 0.998 | 10 | 10.58 | 2.89 | 0.25 | 21 | 2.1 | 68.3 |
| 55 | Altiplano de Teruel | TE | 523 | -0.390 | 0.274 | 0.024 | 17.960 | 15.170 | 1.330 | 0.140 | 0.000 | 0.000 | 0.000 | 1.000 | 13 | 13.14 | 2.45 | 0.21 | 20 | 2.1 | 70.5 |
| 56 | Villar del Salz | TE | 11 | -0.114 | 0.388 | 0.034 | 5.440 | 3.170 | 0.278 | 0.680 | 2.000 | 0.000 | 0.000 | 0.998 | 9 | 8.64 | 3.15 | 0.28 | 21 | 5.4 | 46.4 |
| 57 | Aguatón | TE | 15 | -0.127 | 0.385 | 0.034 | 8.830 | 7.080 | 0.621 | 0.436 | 2.000 | 0.000 | 0.000 | 0.998 | 11 | 10.84 | 3.05 | 0.27 | 22 | 2.4 | 67.6 |
| 58 | Orihuela del Tremedal | TE | 1 | 0.039 | 0.457 | 0.040 | 3.190 | 1.390 | 0.122 | 0.948 | 0.000 | 0.000 | 0.000 | 1.000 | 1 | 2.36 | 2.21 | 0.19 | 16 | 1.5 | 47.8 |
| 59 | Pozondón | TE | 40 | -0.301 | 0.339 | 0.030 | 5.150 | 2.910 | 0.255 | 0.804 | 0.000 | 0.000 | 0.000 | 1.000 | 8 | 8.24 | 2.57 | 0.23 | 17 | 1.5 | 51.5 |
| 60 | Gea de Albarracín | TE | 4 | -0.059 | 0.430 | 0.038 | 2.500 | 0.580 | 0.051 | 0.992 | 0.000 | 0.000 | 0.000 | 1.000 | 3 | 2.94 | 1.81 | 0.16 | 14 | 8.5 | 72.0 |
| 61 | Celadas Oeste | TE | 2 | 0.145 | 0.512 | 0.045 | 3.130 | 1.300 | 0.114 | 0.952 | 0.000 | 0.000 | 0.000 | 1.000 | 2 | 3.08 | 2.32 | 0.20 | 16 | 2.8 | 80.2 |
| 62 | Celadas Este | TE | 9 | -0.094 | 0.414 | 0.036 | 5.570 | 3.660 | 0.321 | 0.760 | 0.000 | 0.000 | 0.000 | 1.000 | 8 | 8.00 | 2.98 | 0.26 | 19 | 2.8 | 85.3 |
| 63 | Valdecebro | TE | 1 | 0.087 | 0.438 | 0.038 | 2.780 | 1.090 | 0.096 | 0.982 | 0.000 | 0.000 | 0.000 | 1.000 | 1 | 1.63 | 1.55 | 0.14 | 14 | 5.5 | 95.3 |
| 64 | Allepuz | TE | 6 | -0.034 | 0.466 | 0.041 | 8.070 | 5.850 | 0.513 | 0.498 | 6.000 | 0.000 | 0.000 | 0.998 | 10 | 10.24 | 3.38 | 0.30 | 22 | 3.1 | 111.5 |
| 65 | Alagón | Z | 1 | 0.103 | 0.471 | 0.041 | 2.250 | 0.460 | 0.040 | 0.984 | 0.000 | 0.000 | 0.000 | 1.000 | 1 | 1.28 | 0.88 | 0.08 | 11 | 8.0 | 125.8 |
| 66 | Utebo | Z | 1 | 0.125 | 0.443 | 0.039 | 2.000 | 0.000 | 0.000 | 0.996 | 0.000 | 0.000 | 0.000 | 1.000 | 1 | 1.68 | 1.34 | 0.12 | 12 | 0.8 | 125.1 |
| 67 | Juslibol | Z | 3 | 0.040 | 0.455 | 0.040 | 3.600 | 1.570 | 0.138 | 0.960 | 0.000 | 0.000 | 0.000 | 1.000 | 3 | 3.62 | 2.50 | 0.22 | 17 | 0.8 | 126.5 |
| 68 | Lumpiaque | Z | 3 | 0.045 | 0.464 | 0.041 | 3.140 | 1.850 | 0.162 | 0.942 | 0.000 | 0.000 | 0.000 | 1.000 | 3 | 3.84 | 2.80 | 0.25 | 18 | 4.1 | 99.9 |
| 69 | Val de Urrea | Z | 104 | -0.307 | 0.302 | 0.026 | 10.380 | 7.960 | 0.698 | 0.442 | 0.000 | 0.000 | 0.000 | 1.000 | 11 | 10.96 | 2.72 | 0.24 | 20 | 4.1 | 101.0 |
| 70 | Longares-Mezalocha | Z | 13 | -0.198 | 0.382 | 0.034 | 4.980 | 3.510 | 0.308 | 0.908 | 0.000 | 0.000 | 0.000 | 1.000 | 6 | 6.29 | 2.69 | 0.24 | 18 | 11.4 | 90.6 |
| 71 | Cuarte de Huerva | Z | 4 | 0.011 | 0.492 | 0.043 | 4.580 | 2.510 | 0.220 | 0.774 | 0.000 | 0.000 | 0.000 | 1.000 | 7 | 7.33 | 3.09 | 0.27 | 17 | 0.3 | 114.6 |
| 72 | Belchite | Z | 189 | -0.380 | 0.300 | 0.026 | 8.260 | 5.390 | 0.472 | 0.508 | 0.000 | 0.000 | 0.000 | 1.000 | 10 | 10.69 | 2.43 | 0.21 | 17 | 0.3 | 111.1 |
| 73 | Monegros | Z | 140 | -0.322 | 0.293 | 0.026 | 11.880 | 10.910 | 0.956 | 0.332 | 0.000 | 0.000 | 0.000 | 1.000 | 11 | 11.67 | 2.61 | 0.23 | 20 | 1.0 | 141.9 |
| 74 | Gelsa | Z | 14 | -0.169 | 0.385 | 0.034 | 4.890 | 4.210 | 0.369 | 0.806 | 0.000 | 0.000 | 0.000 | 1.000 | 8 | 7.69 | 2.83 | 0.25 | 17 | 1.0 | 147.0 |
| 75 | Laguna de Pito | Z | 1 | 0.093 | 0.420 | 0.037 | 2.000 | 0.000 | 0.000 | 0.998 | 0.000 | 0.000 | 0.000 | 1.000 | 1 | 1.37 | 1.08 | 0.09 | 13 | 0.1 | 164.2 |
| 76 | Bujaraloz | Z | 1 | 0.125 | 0.473 | 0.041 | 2.500 | 0.710 | 0.062 | 0.996 | 0.000 | 0.000 | 0.000 | 1.000 | 1 | 1.16 | 0.61 | 0.05 | 11 | 0.1 | 168.9 |
| 77 | Alforque | Z | 2 | 0.151 | 0.517 | 0.045 | 3.240 | 0.970 | 0.085 | 0.966 | 0.000 | 0.000 | 0.000 | 1.000 | 3 | 3.41 | 2.49 | 0.22 | 14 | 2.0 | 143.6 |
| 78 | Azaila | TE | 3 | 0.049 | 0.477 | 0.042 | 3.480 | 2.080 | 0.182 | 0.934 | 0.000 | 0.000 | 0.000 | 1.000 | 4 | 4.42 | 2.82 | 0.25 | 14 | 0.1 | 130.0 |
| 79 | Vinaceite | TE | 1 | -0.005 | 0.445 | 0.039 | 3.500 | 1.810 | 0.159 | 0.936 | 0.000 | 0.000 | 0.000 | 1.000 | 3 | 3.88 | 2.92 | 0.26 | 17 | 0.1 | 124.9 |
| 80 | Lagata | Z | 1 | -0.010 | 0.450 | 0.039 | 5.330 | 4.080 | 0.358 | 0.742 | 0.000 | 0.000 | 0.000 | 1.000 | 5 | 5.42 | 4.00 | 0.35 | 20 | 3.9 | 103.9 |
| 81 | Lécera | Z | 41 | -0.261 | 0.336 | 0.029 | 5.980 | 4.110 | 0.360 | 0.622 | 0.000 | 0.000 | 0.000 | 1.000 | 9 | 9.50 | 2.83 | 0.25 | 20 | 3.9 | 111.6 |
| 82 | Albalate del Arzobispo | TE | 29 | -0.277 | 0.336 | 0.029 | 4.820 | 3.100 | 0.272 | 0.844 | 0.000 | 0.000 | 0.000 | 1.000 | 8 | 7.77 | 2.50 | 0.22 | 18 | 6.1 | 124.5 |
| 83 | Molinos | TE | 39 | -0.268 | 0.339 | 0.030 | 7.210 | 5.200 | 0.456 | 0.716 | 0.000 | 0.000 | 0.000 | 1.000 | 9 | 9.06 | 2.77 | 0.24 | 17 | 21.7 | 125.5 |
| 84 | Saelices | CU | 3 | -0.019 | 0.351 | 0.031 | 0.000 | 0.000 | 0.000 | 1.000 | 0.000 | 0.000 | 0.000 | 1.000 | 2 | 2.06 | 1.30 | 0.11 | 8 | 17.9 | 118.4 |
| 85 | Zafra de Záncara | CU | 5 | -0.171 | 0.401 | 0.035 | 4.000 | 0.000 | 0.000 | 0.998 | 0.000 | 0.000 | 0.000 | 1.000 | 2 | 2.70 | 1.47 | 0.13 | 11 | 17.9 | 117.8 |
| 86 | Valeria | CU | 24 | -0.276 | 0.351 | 0.031 | 3.910 | 1.940 | 0.170 | 0.914 | 0.000 | 0.000 | 0.000 | 1.000 | 7 | 6.88 | 2.42 | 0.21 | 17 | 25.8 | 116.1 |
| 87 | Carboneras de Guadazaón | CU | 9 | -0.241 | 0.391 | 0.034 | 2.500 | 0.710 | 0.062 | 0.996 | 0.000 | 0.000 | 0.000 | 1.000 | 4 | 4.03 | 1.94 | 0.17 | 11 | 9.7 | 103.9 |
| 88 | Cardenete | CU | 9 | -0.141 | 0.379 | 0.033 | 0.000 | 0.000 | 0.000 | 1.000 | 0.000 | 0.000 | 0.000 | 1.000 | 2 | 2.36 | 1.28 | 0.11 | 7 | 9.7 | 118.6 |
| 89 | Moya | CU | 83 | -0.277 | 0.314 | 0.028 | 11.510 | 9.550 | 0.837 | 0.368 | 4.000 | 1.410 | 0.124 | 0.996 | 12 | 11.57 | 3.03 | 0.27 | 22 | 0.1 | 109.8 |
| 90 | Ademuz | V | 85 | -0.283 | 0.305 | 0.027 | 11.030 | 8.800 | 0.771 | 0.428 | 3.330 | 0.580 | 0.051 | 0.994 | 11 | 11.36 | 2.97 | 0.26 | 23 | 0.1 | 97.9 |
| 91 | Sierra de Javalambre | TE | 61 | -0.266 | 0.322 | 0.028 | 8.120 | 5.720 | 0.501 | 0.532 | 0.000 | 0.000 | 0.000 | 1.000 | 10 | 10.31 | 2.83 | 0.25 | 20 | 7.9 | 104.9 |
| 92 | Hoya Gonzalo | AB | 5 | -0.155 | 0.407 | 0.036 | 5.000 | 0.000 | 0.000 | 0.998 | 0.000 | 0.000 | 0.000 | 1.000 | 2 | 2.76 | 1.52 | 0.13 | 12 | 42.4 | 208.6 |
| 93 | Herrada del Manco | MU | 1 | 0.000 | 0.000 | 0.000 | 0.000 | 0.000 | 0.000 | 1.000 | 0.000 | 0.000 | 0.000 | 1.000 | 1 | 1.00 | 0.00 | 0.00 | 1 | 3.2 | 260.5 |
| 94 | Moratillas | MU | 0 | 0.000 | 0.000 | 0.000 | 0.000 | 0.000 | 0.000 | 1.000 | 0.000 | 0.000 | 0.000 | 1.000 | 1 | 1.00 | 0.00 | 0.00 | 1 | 3.2 | 254.8 |
| 95 | Sierra del Picarcho | MU | 14 | -0.275 | 0.378 | 0.033 | 3.440 | 1.890 | 0.166 | 0.964 | 0.000 | 0.000 | 0.000 | 1.000 | 5 | 5.31 | 2.25 | 0.20 | 12 | 38.2 | 281.5 |
| 96 | Padul | GR | 3 | -0.018 | 0.365 | 0.032 | 0.000 | 0.000 | 0.000 | 1.000 | 0.000 | 0.000 | 0.000 | 1.000 | 1 | 1.87 | 1.15 | 0.10 | 7 | 84.8 | 449.1 |
| 97 | LLano de Los Brincos-La Mota | AL | 1 | 0.000 | 0.000 | 0.000 | 0.000 | 0.000 | 0.000 | 1.000 | 0.000 | 0.000 | 0.000 | 1.000 | 1 | 1.00 | 0.04 | 0.00 | 2 | 29.4 | 440.4 |
| 98 | Los Sebastianes | AL | 4 | -0.109 | 0.402 | 0.035 | 0.000 | 0.000 | 0.000 | 1.000 | 0.000 | 0.000 | 0.000 | 1.000 | 2 | 1.67 | 0.58 | 0.05 | 10 | 7.2 | 413.2 |
| 99 | Karst de Sorbas | AL | 6 | -0.198 | 0.415 | 0.036 | 0.000 | 0.000 | 0.000 | 1.000 | 0.000 | 0.000 | 0.000 | 1.000 | 3 | 3.24 | 1.61 | 0.14 | 10 | 7.2 | 414.9 |
| 100 | Las Amoladeras | AL | 17 | -0.284 | 0.375 | 0.033 | 3.920 | 2.750 | 0.241 | 0.976 | 0.000 | 0.000 | 0.000 | 1.000 | 6 | 5.71 | 2.25 | 0.20 | 15 | 25.9 | 446.5 |
|  | **Metapopulation** |  | **5167** | **-0.401** | **0.246** | **0.022** | **311.520** | **198.480** | **17.398** | **0.000** | **8.060** | **6.110** | **0.536** | **0.842** | **18** | **19.07** | **2.50** | **0.22** | **27** |  |  |

**Supplementary Table S2**. Critical areas: subpopulations of a higher concern regarding their imminent extinction (ordered by ascendant *T_mean_*) or their strong population decline (ordered by ascendant *r*). Estimated initial population size in 2020 (*N_0_*); mean population growth rate (*r*) with standard deviation (*SD_r_*) and 95% confidence interval (*CI_r_*) across 500 iterations; mean population size in *t* years (*N_t_*); probability of extinction in *t* years (*P_0_(t)*: equivalent to the proportion of the 500 iterations in which the population is extinct or remains extant); *T_med_*: median time to extinction of the 500 iterations; *T_mean_*: mean time to extinction of the 500 iterations; *T_max_*: maximum time to extinction (equivalent to the year in which all 500 iterations result in metapopulation is extinction); *Dist. N*: linear distance to the closest neighbour subpopulation (border to border); *Dist. C*: linear distance to the metapopulation centroid.

| **ID / Subpopulation** | | **Prov** | ***N_0_*** | ***r*** | ***SD(r)*** | ***CI(r)*** | ***N_10_*** | ***SD(N_10_)*** | ***CI(N_10_)*** | ***P_0_(10)*** | ***N_20_*** | ***SD(N_20_)*** | ***CI(N_20_)*** | ***P_0_(20)*** | ***T_med_*** | ***T_mean_*** | ***SD(T_mean_)*** | ***CI(T_mean_)*** | ***T_max_*** | ***Dist. N*** | ***Dist. C*** |
| --- | --- | --- | --- | --- | --- | --- | --- | --- | --- | --- | --- | --- | --- | --- | --- | --- | --- | --- | --- | --- | --- |
| ***Imminent extinction*** | |  |  |  |  |  |  |  |  |  |  |  |  |  |  |  |  |  |  |  |  |
| 97 | Llano de Los Brincos-La Mota | AL | 1 | 0.000 | 0.000 | 0.000 | 0.000 | 0.000 | 0.000 | 1.000 | 0.000 | 0.000 | 0.000 | 1.000 | 1 | 1.00 | 0.04 | 0.00 | 2 | 29.4 | 440.4 |
| 94 | Moratillas | MU | 0 | 0.000 | 0.000 | 0.000 | 0.000 | 0.000 | 0.000 | 1.000 | 0.000 | 0.000 | 0.000 | 1.000 | 1 | 1.00 | 0.00 | 0.00 | 1 | 3.2 | 254.8 |
| 93 | Herrada del Manco | MU | 1 | 0.000 | 0.000 | 0.000 | 0.000 | 0.000 | 0.000 | 1.000 | 0.000 | 0.000 | 0.000 | 1.000 | 1 | 1.00 | 0.00 | 0.00 | 1 | 3.2 | 260.5 |
| 3 | Villanueva de Valrojo | ZA | 1 | 0.000 | 0.000 | 0.000 | 0.000 | 0.000 | 0.000 | 1.000 | 0.000 | 0.000 | 0.000 | 1.000 | 1 | 1.00 | 0.04 | 0.00 | 3 | 14.0 | 368.3 |
| 2 | Donadillo | ZA | 1 | 0.000 | 0.000 | 0.000 | 0.000 | 0.000 | 0.000 | 1.000 | 0.000 | 0.000 | 0.000 | 1.000 | 1 | 1.00 | 0.00 | 0.00 | 1 | 14.0 | 382.0 |
| 5 | Vegalatrave | ZA | 1 | 0.269 | 0.367 | 0.032 | 0.000 | 0.000 | 0.000 | 1.000 | 0.000 | 0.000 | 0.000 | 1.000 | 1 | 1.04 | 0.28 | 0.02 | 7 | 9.5 | 350.7 |
| 18 | Talamantes | Z | 1 | 0.038 | 0.363 | 0.032 | 2.000 | 0.000 | 0.000 | 0.998 | 0.000 | 0.000 | 0.000 | 1.000 | 1 | 1.05 | 0.40 | 0.04 | 11 | 17.1 | 97.8 |
| 8 | Velilla de la Sierra | SO | 1 | 0.190 | 0.323 | 0.028 | 0.000 | 0.000 | 0.000 | 1.000 | 0.000 | 0.000 | 0.000 | 1.000 | 1 | 1.07 | 0.37 | 0.03 | 6 | 3.7 | 106.0 |
| 11 | Pozalmuro | SO | 1 | 0.118 | 0.311 | 0.027 | 0.000 | 0.000 | 0.000 | 1.000 | 0.000 | 0.000 | 0.000 | 1.000 | 1 | 1.09 | 0.45 | 0.04 | 7 | 0.1 | 96.4 |
| 76 | Bujaraloz | Z | 1 | 0.125 | 0.473 | 0.041 | 2.500 | 0.710 | 0.062 | 0.996 | 0.000 | 0.000 | 0.000 | 1.000 | 1 | 1.16 | 0.61 | 0.05 | 11 | 0.1 | 168.9 |
| 65 | Alagón | Z | 1 | 0.103 | 0.471 | 0.041 | 2.250 | 0.460 | 0.040 | 0.984 | 0.000 | 0.000 | 0.000 | 1.000 | 1 | 1.28 | 0.88 | 0.08 | 11 | 8.0 | 125.8 |
| 75 | Laguna de Pito | Z | 1 | 0.093 | 0.420 | 0.037 | 2.000 | 0.000 | 0.000 | 0.998 | 0.000 | 0.000 | 0.000 | 1.000 | 1 | 1.37 | 1.08 | 0.09 | 13 | 0.1 | 164.2 |
| 15 | Deza | SO | 1 | 0.108 | 0.418 | 0.037 | 3.000 | 0.000 | 0.000 | 0.996 | 0.000 | 0.000 | 0.000 | 1.000 | 1 | 1.40 | 1.08 | 0.09 | 14 | 5.3 | 60.9 |
| 63 | Valdecebro | TE | 1 | 0.087 | 0.438 | 0.038 | 2.780 | 1.090 | 0.096 | 0.982 | 0.000 | 0.000 | 0.000 | 1.000 | 1 | 1.63 | 1.55 | 0.14 | 14 | 5.5 | 95.3 |
| 98 | Los Sebastianes | AL | 4 | -0.109 | 0.402 | 0.035 | 0.000 | 0.000 | 0.000 | 1.000 | 0.000 | 0.000 | 0.000 | 1.000 | 2 | 1.67 | 0.58 | 0.05 | 10 | 7.2 | 413.2 |
| 66 | Utebo | Z | 1 | 0.125 | 0.443 | 0.039 | 2.000 | 0.000 | 0.000 | 0.996 | 0.000 | 0.000 | 0.000 | 1.000 | 1 | 1.68 | 1.34 | 0.12 | 12 | 0.8 | 125.1 |
| 9 | Arancón | SO | 2 | 0.175 | 0.384 | 0.034 | 0.000 | 0.000 | 0.000 | 1.000 | 0.000 | 0.000 | 0.000 | 1.000 | 1 | 1.69 | 1.04 | 0.09 | 6 | 2.4 | 100.8 |
| 42 | Alhama de Aragón | Z | 2 | 0.340 | 0.436 | 0.038 | 2.130 | 0.350 | 0.031 | 0.984 | 0.000 | 0.000 | 0.000 | 1.000 | 1 | 1.78 | 1.08 | 0.09 | 12 | 7.6 | 42.4 |
| 96 | Padul | GR | 3 | -0.018 | 0.365 | 0.032 | 0.000 | 0.000 | 0.000 | 1.000 | 0.000 | 0.000 | 0.000 | 1.000 | 1 | 1.87 | 1.15 | 0.10 | 7 | 84.8 | 449.1 |
| ***Fastest population decline*** | |  |  |  |  |  |  |  |  |  |  |  |  |  |  |  |  |  |  |  |  |
| 36 | Layna | SO | 761 | -0.453 | 0.269 | 0.024 | 11.250 | 7.640 | 0.670 | 0.196 | 0.000 | 0.000 | 0.000 | 1.000 | 12 | 12.21 | 2.11 | 0.18 | 19 | 0.2 | 27.5 |
| 32 | Altos de Barahona | SO | 1125 | -0.448 | 0.263 | 0.023 | 17.680 | 13.380 | 1.173 | 0.086 | 4.000 | 0.000 | 0.000 | 0.998 | 13 | 13.27 | 2.17 | 0.19 | 21 | 0.1 | 71.6 |
| 55 | Altiplano de Teruel | TE | 523 | -0.390 | 0.274 | 0.024 | 17.960 | 15.170 | 1.330 | 0.140 | 0.000 | 0.000 | 0.000 | 1.000 | 13 | 13.14 | 2.45 | 0.21 | 20 | 2.1 | 70.5 |
| 72 | Belchite | Z | 189 | -0.380 | 0.300 | 0.026 | 8.260 | 5.390 | 0.472 | 0.508 | 0.000 | 0.000 | 0.000 | 1.000 | 10 | 10.69 | 2.43 | 0.21 | 17 | 0.3 | 111.1 |
| 44 | Paramera de Molina | GU | 233 | -0.350 | 0.291 | 0.025 | 11.700 | 8.800 | 0.771 | 0.198 | 2.000 | 0.000 | 0.000 | 0.998 | 12 | 12.33 | 2.51 | 0.22 | 21 | 0.1 | 10.2 |
| 73 | Monegros | Z | 140 | -0.322 | 0.293 | 0.026 | 11.880 | 10.910 | 0.956 | 0.332 | 0.000 | 0.000 | 0.000 | 1.000 | 11 | 11.67 | 2.61 | 0.23 | 20 | 1.0 | 141.9 |
| 53 | Blancas | TE | 253 | -0.319 | 0.284 | 0.025 | 20.580 | 17.160 | 1.504 | 0.134 | 4.330 | 3.010 | 0.264 | 0.988 | 14 | 13.62 | 2.89 | 0.25 | 22 | 3.2 | 31.6 |
| 69 | Val de Urrea | Z | 104 | -0.307 | 0.302 | 0.026 | 10.380 | 7.960 | 0.698 | 0.442 | 0.000 | 0.000 | 0.000 | 1.000 | 11 | 10.96 | 2.72 | 0.24 | 20 | 4.1 | 101.0 |
| 52 | Río Gallo | GU | 133 | -0.306 | 0.295 | 0.026 | 11.590 | 8.860 | 0.777 | 0.270 | 4.670 | 1.530 | 0.134 | 0.994 | 12 | 12.08 | 2.83 | 0.25 | 24 | 0.2 | 16.5 |
| 25 | Hoces del Duratón | SG | 94 | -0.305 | 0.305 | 0.027 | 10.740 | 8.550 | 0.749 | 0.460 | 2.000 | 0.000 | 0.000 | 0.998 | 11 | 10.94 | 2.76 | 0.24 | 21 | 1.0 | 154.1 |

**Supplementary Table S3.** Results of the sensitivity analysis in the probability of extinction in 20 years *P_0_(20)*, population growth (*r*) and time to extinction (*T_median_*, *T_mean_* and *T_max_*. Bold values represent the base model. See also Supplementary Figs. S1-S3.

| **Parameter** | ***r*** | ***P_0_(20)*** | ***T_median_*** | ***T_mean_*** | ***T_max_*** |
| --- | --- | --- | --- | --- | --- |
| *Productivity (offspring/brood)* |  |  |  |  |  |
| 1.2 | -0.52 | 1.000 | 14 | 15.2 | 20 |
| 1.3 | -0.483 | 0.996 | 15 | 16.3 | 24 |
| 1.4 | -0.437 | 0.968 | 17 | 17.7 | 24 |
| **1.5** | **-0.401** | **0.842** | **18** | **19.1** | **27** |
| 1.6 | -0.365 | 0.606 | 20 | 21 | 27 |
| 1.7 | -0.326 | 0.330 | 22 | 23 | 28 |
| 1.8 | -0.283 | 0.136 | 24 | 24.9 | 28 |
| 1.9 | -0.236 | 0.036 | 27 | 26.8 | 29 |
| *Breeding females* |  |  |  |  |  |
| 75 | -0.526 | 1.000 | 14 | 15 | 20 |
| 80 | -0.499 | 0.996 | 15 | 15.6 | 21 |
| 85 | -0.472 | 0.982 | 15 | 16.5 | 24 |
| 90 | -0.441 | 0.950 | 16 | 17.5 | 25 |
| 95 | -0.418 | 0.900 | 17 | 18.5 | 24 |
| **100** | **-0.401** | **0.842** | **18** | **19.1** | **27** |
| *Male survival* |  |  |  |  |  |
| 30 | -0.555 | 1.000 | 14 | 14.6 | 20 |
| 40 | -0.455 | 0.976 | 16 | 17.3 | 23 |
| **48** | **-0.401** | **0.842** | **18** | **19.1** | **27** |
| 50 | -0.396 | 0.848 | 18 | 19.4 | 27 |
| 60 | -0.354 | 0.650 | 20 | 20.8 | 27 |
| 70 | -0.327 | 0.522 | 20 | 21.6 | 27 |
| 80 | -0.312 | 0.466 | 21 | 21.9 | 27 |
| 90 | -0.295 | 0.444 | 21 | 22.1 | 28 |
| 100 | -0.287 | 0.454 | 21 | 22.1 | 28 |
| *Female survival* |  |  |  |  |  |
| 20 | -0.509 | 1.000 | 14 | 14.9 | 20 |
| 30 | -0.411 | 0.902 | 18 | 18.7 | 25 |
| **31** | **-0.401** | **0.842** | **18** | **19.1** | **27** |
| 40 | -0.339 | 0.370 | 21 | 22.5 | 27 |
| 50 | -0.291 | 0.074 | 25 | 25.3 | 28 |
| 60 | -0.264 | 0.008 | 26 | 26.6 | 28 |
| *Juvenile survival* |  |  |  |  |  |
| 25 | -0.519 | 1.000 | 14 | 15.2 | 19 |
| 27.5 | -0.467 | 0.986 | 16 | 16.8 | 23 |
| 30 | -0.425 | 0.930 | 17 | 18.1 | 25 |
| **31** | **-0.401** | **0.842** | **18** | **19.1** | **27** |
| 32.5 | -0.373 | 0.696 | 19 | 20.5 | 27 |
| 35 | -0.324 | 0.334 | 22 | 22.9 | 27 |
| 37.5 | -0.271 | 0.056 | 25 | 25.8 | 29 |
| 40 | -0.21 | 0.000 | 27 | 27.5 | 28 |
| *Dispersal survival* |  |  |  |  |  |
| 0 | -0.443 | 0.944 | 16 | 17.6 | 26 |
| 10 | -0.434 | 0.918 | 17 | 18 | 25 |
| 20 | -0.427 | 0.948 | 17 | 18.2 | 26 |
| 30 | -0.424 | 0.904 | 17 | 18.3 | 27 |
| 40 | -0.415 | 0.898 | 17 | 18.6 | 27 |
| **50** | **-0.401** | **0.842** | **18** | **19.1** | **27** |
| 60 | -0.391 | 0.818 | 18 | 19.5 | 27 |
| 70 | -0.378 | 0.714 | 19 | 20.1 | 26 |
| 80 | -0.368 | 0.718 | 19 | 20.4 | 27 |
| 90 | -0.352 | 0.524 | 20 | 21.4 | 27 |
| 100 | -0.337 | 0.448 | 21 | 21.8 | 27 |

**Supplementary Table S4**. Effects of the habitat management simulation in the metapopulation under nine different spatial/temporal scenarios, varying the number of subpopulations managed (n = 5, 10, 15) and number of years of management (n = 3, 5,10).

| **Scenario** | **Nº subpop.** | **Nº years** | ***r*** | ***SD(r)*** | ***CI(r)*** | ***P_0_(20)*** | ***T_med_*** | ***T_mean_*** | ***SD(T_mean_)*** | ***CI(T_mean_)*** | ***T_max_*** |
| --- | --- | --- | --- | --- | --- | --- | --- | --- | --- | --- | --- |
| No management | 0 | 0 | -0.401 | 0.246 | 0.022 | 0.842 | 18 | 19.07 | 2.50 | 0.22 | 27 |
| Scenario 1 | 5 | 3 | -0.381 | 0.247 | 0.022 | 0.750 | 19 | 19.97 | 2.43 | 0.21 | 29 |
| Scenario 2 | 5 | 5 | -0.380 | 0.247 | 0.022 | 0.750 | 19 | 20.03 | 2.28 | 0.20 | 26 |
| Scenario 3 | 5 | 10 | -0.377 | 0.232 | 0.020 | 0.774 | 19 | 20.10 | 1.92 | 0.17 | 26 |
| Scenario 4 | 10 | 3 | -0.365 | 0.246 | 0.022 | 0.618 | 20 | 20.92 | 2.70 | 0.24 | 33 |
| Scenario 5 | 10 | 5 | -0.367 | 0.249 | 0.022 | 0.654 | 19 | 20.76 | 2.84 | 0.25 | 31 |
| Scenario 6 | 10 | 10 | -0.357 | 0.232 | 0.020 | 0.622 | 20 | 21.19 | 2.63 | 0.23 | 34 |
| Scenario 7 | 15 | 3 | -0.343 | 0.231 | 0.020 | 0.482 | 21 | 21.95 | 2.91 | 0.26 | 31 |
| Scenario 8 | 15 | 5 | -0.348 | 0.236 | 0.021 | 0.522 | 20 | 21.64 | 2.87 | 0.25 | 33 |
| Scenario 9 | 15 | 10 | -0.340 | 0.225 | 0.020 | 0.500 | 20 | 21.98 | 3.16 | 0.28 | 46 |

**Supplementary Table S5**. Effects in the metapopulation parameters of different intensities of habitat management, with step increases in productivity and carrying capacity (K): 1, 5, 10, 15 and 20%. All simulations based on scenario 4 (subset of 10 subpopulations managed during 3 consecutive years; Supplementary Table S4).

| Scenario | *r* | *SD(r)* | *CI(r)* | *P_0_(20)* | *T_med_* | *T_mean_* | *SD(T_mean_)* | *CI(T_mean_)* | *T_max_* |
| --- | --- | --- | --- | --- | --- | --- | --- | --- | --- |
| No management | -0.401 | 0.246 | 0.022 | 0.842 | 18 | 19.07 | 2.50 | 0.22 | 27 |
| Prod./K increase 1% | -0.400 | 0.247 | 0.022 | 0.860 | 18 | 19.15 | 2.20 | 0.19 | 27 |
| Prod./K increase 5% | -0.387 | 0.249 | 0.022 | 0.782 | 19 | 19.73 | 2.48 | 0.22 | 31 |
| Prod./K increase 10% | -0.365 | 0.246 | 0.022 | 0.618 | 20 | 20.92 | 2.70 | 0.24 | 33 |
| Prod./K increase 15% | -0.339 | 0.242 | 0.021 | 0.436 | 21 | 22.24 | 3.08 | 0.27 | 34 |
| Prod./K increase 20% | -0.311 | 0.243 | 0.021 | 0.240 | 23 | 24.2 | 4.03 | 0.35 | 42 |

**Supplementary Table S6.** Results of the simulation of individuals translocation. Donor subpopulations were those with more than 100 males (n=7). Recipient subpopulations were those with the most unfavorable situation in the PVA results (shortest mean time to extinction), two scenarios were simulated: seven recipient subpopulations close to the metapopulation core and seven recipients distant to the core. For each scenario, six different harvest alternatives are offered: 1+1, 2+2, 3+3, 5+5, 10+10 and 10+6 males/females, this last one adjusted to the species sex ratio. In all of them, movements were carried out during 3 consecutive years. Harvested individuals were introduced randomly in recipient subpopulations.

| **ID** | **Donor subpopulations** | **Harvesting** | ***r*** | ***CI(r)*** | ***P_0_(10)*** | ***P_0_(20)*** | ***T_mean_*** | ***CI(T_mean_)*** | ***T_max_*** |
| --- | --- | --- | --- | --- | --- | --- | --- | --- | --- |
| 32 | Altos de Barahona | Before translocation | -0.448 | 0.023 | 0.086 | 0.998 | 13.27 | 0.19 | 21 |
| 36 | Layna | Before translocation | -0.453 | 0.024 | 0.196 | 1.000 | 12.21 | 0.18 | 19 |
| 44 | Paramera de Molina | Before translocation | -0.350 | 0.025 | 0.198 | 0.998 | 12.33 | 0.22 | 21 |
| 48 | Segura de los Baños | Before translocation | -0.297 | 0.025 | 0.098 | 0.972 | 14.03 | 0.26 | 27 |
| 53 | Blancas | Before translocation | -0.319 | 0.025 | 0.134 | 0.988 | 13.62 | 0.25 | 22 |
| 55 | Altiplano de Teruel | Before translocation | -0.390 | 0.024 | 0.140 | 1.000 | 13.14 | 0.21 | 20 |
| 72 | Belchite | Before translocation | -0.380 | 0.026 | 0.508 | 1.000 | 10.69 | 0.21 | 17 |
| 32 | Altos de Barahona | 1M/1F | -0.443 | 0.023 | 0.084 | 1.000 | 13.40 | 0.19 | 20 |
| 36 | Layna | 1M/1F | -0.447 | 0.024 | 0.178 | 1.000 | 12.30 | 0.18 | 19 |
| 44 | Paramera de Molina | 1M/1F | -0.350 | 0.025 | 0.266 | 0.998 | 12.10 | 0.23 | 22 |
| 48 | Segura de los Baños | 1M/1F | -0.306 | 0.026 | 0.130 | 0.984 | 13.70 | 0.25 | 24 |
| 53 | Blancas | 1M/1F | -0.323 | 0.025 | 0.144 | 0.994 | 13.40 | 0.24 | 22 |
| 55 | Altiplano de Teruel | 1M/1F | -0.389 | 0.024 | 0.104 | 0.996 | 13.30 | 0.22 | 22 |
| 72 | Belchite | 1M/1F | -0.397 | 0.026 | 0.542 | 1.000 | 10.20 | 0.19 | 19 |
| 32 | Altos de Barahona | 2M/2F | -0.445 | 0.023 | 0.100 | 0.996 | 13.30 | 0.20 | 21 |
| 36 | Layna | 2M/2F | -0.453 | 0.024 | 0.192 | 1.000 | 12.10 | 0.19 | 19 |
| 44 | Paramera de Molina | 2M/2F | -0.348 | 0.025 | 0.270 | 0.996 | 12.20 | 0.22 | 21 |
| 48 | Segura de los Baños | 2M/2F | -0.309 | 0.025 | 0.120 | 0.984 | 13.70 | 0.25 | 25 |
| 53 | Blancas | 2M/2F | -0.326 | 0.025 | 0.174 | 0.996 | 13.30 | 0.25 | 21 |
| 55 | Altiplano de Teruel | 2M/2F | -0.396 | 0.024 | 0.142 | 0.994 | 13.00 | 0.23 | 23 |
| 72 | Belchite | 2M/2F | -0.400 | 0.026 | 0.522 | 1.000 | 10.30 | 0.20 | 17 |
| 32 | Altos de Barahona | 3M/3F | -0.450 | 0.024 | 0.100 | 0.996 | 13.09 | 0.19 | 21 |
| 36 | Layna | 3M/3F | -0.450 | 0.024 | 0.210 | 1.000 | 12.01 | 0.18 | 20 |
| 44 | Paramera de Molina | 3M/3F | -0.360 | 0.025 | 0.260 | 0.996 | 11.96 | 0.21 | 23 |
| 48 | Segura de los Baños | 3M/3F | -0.310 | 0.025 | 0.160 | 0.982 | 13.32 | 0.27 | 24 |
| 53 | Blancas | 3M/3F | -0.330 | 0.025 | 0.170 | 0.992 | 13.04 | 0.24 | 22 |
| 55 | Altiplano de Teruel | 3M/3F | -0.390 | 0.025 | 0.140 | 0.996 | 13.08 | 0.22 | 23 |
| 72 | Belchite | 3M/3F | -0.400 | 0.026 | 0.530 | 1.000 | 10.30 | 0.21 | 17 |
| 32 | Altos de Barahona | 5M/5F | -0.451 | 0.023 | 0.098 | 0.998 | 13.10 | 0.20 | 21 |
| 36 | Layna | 5M/5F | -0.456 | 0.024 | 0.208 | 0.998 | 12.20 | 0.19 | 21 |
| 44 | Paramera de Molina | 5M/5F | -0.366 | 0.026 | 0.318 | 0.998 | 11.70 | 0.23 | 21 |
| 48 | Segura de los Baños | 5M/5F | -0.322 | 0.026 | 0.186 | 0.986 | 13.10 | 0.28 | 25 |
| 53 | Blancas | 5M/5F | -0.338 | 0.025 | 0.174 | 0.994 | 12.70 | 0.25 | 22 |
| 55 | Altiplano de Teruel | 5M/5F | -0.402 | 0.024 | 0.168 | 0.998 | 12.80 | 0.21 | 21 |
| 72 | Belchite | 5M/5F | -0.416 | 0.027 | 0.596 | 1.000 | 9.80 | 0.23 | 20 |
| 32 | Altos de Barahona | 10M/10F | -0.459 | 0.024 | 0.120 | 0.998 | 12.90 | 0.20 | 21 |
| 36 | Layna | 10M/10F | -0.469 | 0.026 | 0.276 | 1.000 | 11.80 | 0.19 | 20 |
| 44 | Paramera de Molina | 10M/10F | -0.401 | 0.029 | 0.488 | 1.000 | 10.30 | 0.29 | 19 |
| 48 | Segura de los Baños | 10M/10F | -0.355 | 0.027 | 0.348 | 0.994 | 11.60 | 0.32 | 24 |
| 53 | Blancas | 10M/10F | -0.374 | 0.027 | 0.340 | 0.998 | 11.50 | 0.29 | 22 |
| 55 | Altiplano de Teruel | 10M/10F | -0.417 | 0.025 | 0.218 | 1.000 | 12.30 | 0.22 | 19 |
| 72 | Belchite | 10M/10F | -0.463 | 0.028 | 0.786 | 1.000 | 7.40 | 0.30 | 17 |
| 32 | Altos de Barahona | 10M/6F | -0.456 | 0.024 | 0.134 | 1.000 | 12.90 | 0.20 | 20 |
| 36 | Layna | 10M/6F | -0.468 | 0.024 | 0.232 | 1.000 | 11.90 | 0.18 | 19 |
| 44 | Paramera de Molina | 10M/6F | -0.387 | 0.029 | 0.400 | 0.998 | 11.00 | 0.23 | 21 |
| 48 | Segura de los Baños | 10M/6F | -0.338 | 0.026 | 0.270 | 0.986 | 12.40 | 0.28 | 23 |
| 53 | Blancas | 10M/6F | -0.354 | 0.027 | 0.246 | 0.994 | 12.30 | 0.27 | 22 |
| 55 | Altiplano de Teruel | 10M/6F | -0.408 | 0.024 | 0.214 | 0.994 | 12.50 | 0.23 | 22 |
| 72 | Belchite | 10M/6F | -0.427 | 0.027 | 0.646 | 1.000 | 9.30 | 0.25 | 16 |
| **ID** | **Recipients close to the core** | **Harvesting** | ***r*** | ***CI(r)*** | ***P_0_(10)*** | ***P_0_(20)*** | ***T_mean_*** | ***CI(T_mean_)*** | ***T_max_*** |
| 12 | Pinilla del Campo | Before translocation | -0.005 | 0.032 | 1.000 | 1.000 | 2.01 | 0.11 | 9 |
| 15 | Deza | Before translocation | 0.108 | 0.037 | 0.996 | 1.000 | 1.40 | 0.09 | 14 |
| 40 | Cueva de la Hoz | Before translocation | 0.387 | 0.047 | 0.954 | 1.000 | 2.99 | 0.17 | 14 |
| 42 | Alhama de Aragón | Before translocation | 0.340 | 0.038 | 0.984 | 1.000 | 1.78 | 0.09 | 12 |
| 58 | Orihuela del Tremedal | Before translocation | 0.039 | 0.040 | 0.948 | 1.000 | 2.36 | 0.19 | 16 |
| 60 | Gea de Albarracín | Before translocation | -0.059 | 0.038 | 0.992 | 1.000 | 2.94 | 0.16 | 14 |
| 61 | Celadas Oeste | Before translocation | 0.145 | 0.045 | 0.952 | 1.000 | 3.08 | 0.20 | 16 |
| 12 | Pinilla del Campo | 1M/1F | 0.070 | 0.041 | 0.994 | 1.000 | 5.50 | 0.12 | 13 |
| 15 | Deza | 1M/1F | 0.107 | 0.049 | 0.984 | 1.000 | 5.90 | 0.15 | 15 |
| 40 | Cueva de la Hoz | 1M/1F | 0.966 | 0.081 | 0.958 | 1.000 | 2.90 | 0.16 | 15 |
| 42 | Alhama de Aragón | 1M/1F | 0.484 | 0.045 | 0.992 | 1.000 | 2.60 | 0.13 | 13 |
| 58 | Orihuela del Tremedal | 1M/1F | 0.154 | 0.057 | 0.920 | 1.000 | 6.70 | 0.18 | 16 |
| 60 | Gea de Albarracín | 1M/1F | 0.011 | 0.039 | 0.974 | 1.000 | 6.30 | 0.16 | 14 |
| 61 | Celadas Oeste | 1M/1F | 0.101 | 0.049 | 0.930 | 1.000 | 6.80 | 0.18 | 15 |
| 12 | Pinilla del Campo | 2M/2F | 0.027 | 0.045 | 0.976 | 1.000 | 6.62 | 0.11 | 12 |
| 15 | Deza | 2M/2F | 0.018 | 0.051 | 0.996 | 1.000 | 7.20 | 0.09 | 12 |
| 40 | Cueva de la Hoz | 2M/2F | 1.016 | 0.097 | 0.942 | 1.000 | 3.00 | 0.16 | 15 |
| 42 | Alhama de Aragón | 2M/2F | 0.603 | 0.054 | 0.994 | 1.000 | 2.50 | 0.10 | 12 |
| 58 | Orihuela del Tremedal | 2M/2F | 0.088 | 0.060 | 0.962 | 1.000 | 7.90 | 0.20 | 16 |
| 60 | Gea de Albarracín | 2M/2F | -0.010 | 0.043 | 0.992 | 1.000 | 7.70 | 0.17 | 16 |
| 61 | Celadas Oeste | 2M/2F | 0.063 | 0.053 | 0.966 | 1.000 | 7.90 | 0.21 | 16 |
| 12 | Pinilla del Campo | 3M/3F | -0.102 | 0.035 | 0.998 | 1.000 | 7.65 | 0.13 | 15 |
| 15 | Deza | 3M/3F | -0.012 | 0.052 | 0.900 | 1.000 | 8.01 | 0.16 | 14 |
| 40 | Cueva de la Hoz | 3M/3F | 1.090 | 0.100 | 0.930 | 1.000 | 2.94 | 0.17 | 16 |
| 42 | Alhama de Aragón | 3M/3F | 0.703 | 0.061 | 0.996 | 1.000 | 2.44 | 0.13 | 16 |
| 58 | Orihuela del Tremedal | 3M/3F | 0.043 | 0.061 | 0.770 | 1.000 | 8.70 | 0.20 | 17 |
| 60 | Gea de Albarracín | 3M/3F | -0.006 | 0.046 | 0.834 | 1.000 | 8.42 | 0.19 | 19 |
| 61 | Celadas Oeste | 3M/3F | 0.044 | 0.056 | 0.776 | 1.000 | 8.67 | 0.19 | 16 |
| 12 | Pinilla del Campo | 5M/5F | -0.031 | 0.053 | 0.802 | 1.000 | 8.80 | 0.18 | 16 |
| 15 | Deza | 5M/5F | -0.027 | 0.053 | 0.802 | 1.000 | 8.80 | 0.18 | 16 |
| 40 | Cueva de la Hoz | 5M/5F | 1.205 | 0.107 | 0.934 | 1.000 | 2.90 | 0.16 | 15 |
| 42 | Alhama de Aragón | 5M/5F | 0.870 | 0.076 | 0.996 | 1.000 | 2.40 | 0.13 | 11 |
| 58 | Orihuela del Tremedal | 5M/5F | 0.011 | 0.064 | 0.576 | 0.998 | 10.20 | 0.21 | 22 |
| 60 | Gea de Albarracín | 5M/5F | -0.017 | 0.049 | 0.592 | 1.000 | 10.10 | 0.21 | 19 |
| 61 | Celadas Oeste | 5M/5F | 0.023 | 0.061 | 0.602 | 1.000 | 10.00 | 0.20 | 19 |
| 12 | Pinilla del Campo | 10M/10F | -0.048 | 0.058 | 0.466 | 1.000 | 10.80 | 0.20 | 19 |
| 15 | Deza | 10M/10F | 0.016 | 0.059 | 0.646 | 1.000 | 8.90 | 0.18 | 15 |
| 40 | Cueva de la Hoz | 10M/10F | 1.404 | 0.121 | 0.954 | 1.000 | 2.80 | 0.16 | 15 |
| 42 | Alhama de Aragón | 10M/10F | 1.190 | 0.094 | 0.996 | 1.000 | 2.50 | 0.14 | 12 |
| 58 | Orihuela del Tremedal | 10M/10F | -0.022 | 0.069 | 0.292 | 0.998 | 11.80 | 0.21 | 21 |
| 60 | Gea de Albarracín | 10M/10F | 0.005 | 0.058 | 0.320 | 1.000 | 11.70 | 0.21 | 20 |
| 61 | Celadas Oeste | 10M/10F | 0.013 | 0.068 | 0.318 | 1.000 | 11.60 | 0.20 | 19 |
| 12 | Pinilla del Campo | 10M/6F | -0.038 | 0.058 | 0.618 | 1.000 | 9.80 | 0.19 | 17 |
| 15 | Deza | 10M/6F | 0.005 | 0.057 | 0.788 | 1.000 | 8.80 | 0.19 | 16 |
| 40 | Cueva de la Hoz | 10M/6F | 1.309 | 0.115 | 0.956 | 1.000 | 2.80 | 0.16 | 14 |
| 42 | Alhama de Aragón | 10M/6F | 1.095 | 0.088 | 0.996 | 1.000 | 2.50 | 0.14 | 13 |
| 58 | Orihuela del Tremedal | 10M/6F | -0.001 | 0.070 | 0.448 | 0.998 | 10.90 | 0.20 | 21 |
| 60 | Gea de Albarracín | 10M/6F | -0.007 | 0.054 | 0.400 | 1.000 | 11.10 | 0.22 | 19 |
| 61 | Celadas Oeste | 10M/6F | 0.016 | 0.067 | 0.706 | 1.000 | 8.53 | 0.26 | 18 |
| **ID** | **Recipients distant to the core** | **Harvesting** | ***r*** | ***CI(r)*** | ***P_0_(10)*** | ***P_0_(20)*** | ***T_mean_*** | ***CI(T_mean_)*** | ***T_max_*** |
| 2 | Donadillo | Before translocation | 0.000 | 0.000 | 1.000 | 1.000 | 1.00 | 0.00 | 1 |
| 3 | Villanueva de Valrojo | Before translocation | 0.000 | 0.000 | 1.000 | 1.000 | 1.00 | 0.00 | 3 |
| 5 | Vegalatrave | Before translocation | 0.269 | 0.032 | 1.000 | 1.000 | 1.04 | 0.02 | 7 |
| 96 | Padul | Before translocation | -0.018 | 0.032 | 1.000 | 1.000 | 1.87 | 0.10 | 7 |
| 97 | LLano de Los Brincos-La Mota | Before translocation | 0.000 | 0.000 | 1.000 | 1.000 | 1.00 | 0.00 | 2 |
| 98 | Los Sebastianes | Before translocation | -0.109 | 0.035 | 1.000 | 1.000 | 1.67 | 0.05 | 10 |
| 99 | Karst de Sorbas | Before translocation | -0.198 | 0.036 | 1.000 | 1.000 | 3.24 | 0.14 | 10 |
| 2 | Donadillo | 1M/1F | 0.128 | 0.043 | 1.000 | 1.000 | 5.20 | 0.12 | 10 |
| 3 | Villanueva de Valrojo | 1M/1F | 0.150 | 0.045 | 0.998 | 1.000 | 5.10 | 0.13 | 11 |
| 5 | Vegalatrave | 1M/1F | 0.215 | 0.045 | 1.000 | 1.000 | 4.40 | 0.14 | 9 |
| 96 | Padul | 1M/1F | 0.067 | 0.040 | 0.986 | 1.000 | 5.70 | 0.14 | 13 |
| 97 | LLano de Los Brincos-La Mota | 1M/1F | 0.113 | 0.044 | 0.994 | 1.000 | 5.40 | 0.12 | 12 |
| 98 | Los Sebastianes | 1M/1F | 0.017 | 0.038 | 0.988 | 1.000 | 5.90 | 0.13 | 11 |
| 99 | Karst de Sorbas | 1M/1F | -0.050 | 0.036 | 0.982 | 1.000 | 6.00 | 0.14 | 13 |
| 2 | Donadillo | 2M/2F | 0.079 | 0.044 | 0.996 | 1.000 | 5.60 | 0.15 | 11 |
| 3 | Villanueva de Valrojo | 2M/2F | 0.107 | 0.043 | 1.000 | 1.000 | 5.40 | 0.15 | 10 |
| 5 | Vegalatrave | 2M/2F | 0.186 | 0.045 | 1.000 | 1.000 | 4.60 | 0.15 | 10 |
| 96 | Padul | 2M/2F | 0.044 | 0.044 | 0.974 | 1.000 | 7.00 | 0.16 | 16 |
| 97 | LLano de Los Brincos-La Mota | 2M/2F | 0.029 | 0.043 | 0.978 | 1.000 | 6.50 | 0.15 | 13 |
| 98 | Los Sebastianes | 2M/2F | -0.016 | 0.043 | 0.926 | 1.000 | 7.50 | 0.16 | 13 |
| 99 | Karst de Sorbas | 2M/2F | -0.025 | 0.039 | 0.956 | 1.000 | 7.10 | 0.16 | 14 |
| 2 | Donadillo | 3M/3F | 0.087 | 0.044 | 0.992 | 1.000 | 5.96 | 0.16 | 13 |
| 3 | Villanueva de Valrojo | 3M/3F | 0.139 | 0.046 | 0.998 | 1.000 | 5.34 | 0.16 | 12 |
| 5 | Vegalatrave | 3M/3F | 0.216 | 0.045 | 1.000 | 1.000 | 4.45 | 0.16 | 10 |
| 96 | Padul | 3M/3F | 0.058 | 0.048 | 0.930 | 1.000 | 7.43 | 0.17 | 14 |
| 97 | LLano de Los Brincos-La Mota | 3M/3F | 0.038 | 0.043 | 0.980 | 1.000 | 6.67 | 0.15 | 13 |
| 98 | Los Sebastianes | 3M/3F | -0.010 | 0.046 | 0.840 | 1.000 | 8.41 | 0.19 | 18 |
| 99 | Karst de Sorbas | 3M/3F | 0.010 | 0.041 | 0.900 | 1.000 | 7.58 | 0.17 | 15 |
| 2 | Donadillo | 5M/5F | 0.149 | 0.049 | 0.984 | 1.000 | 5.90 | 0.17 | 11 |
| 3 | Villanueva de Valrojo | 5M/5F | 0.188 | 0.051 | 0.992 | 1.000 | 5.40 | 0.18 | 12 |
| 5 | Vegalatrave | 5M/5F | 0.298 | 0.055 | 1.000 | 1.000 | 4.50 | 0.17 | 10 |
| 96 | Padul | 5M/5F | 0.119 | 0.055 | 0.940 | 1.000 | 7.40 | 0.17 | 13 |
| 97 | LLano de Los Brincos-La Mota | 5M/5F | 0.069 | 0.045 | 0.964 | 1.000 | 6.80 | 0.17 | 14 |
| 98 | Los Sebastianes | 5M/5F | -0.022 | 0.050 | 0.584 | 1.000 | 10.20 | 0.23 | 20 |
| 99 | Karst de Sorbas | 5M/5F | 0.078 | 0.047 | 0.894 | 1.000 | 7.80 | 0.18 | 17 |
| 2 | Donadillo | 10M/10F | 0.305 | 0.063 | 0.990 | 1.000 | 5.80 | 0.18 | 12 |
| 3 | Villanueva de Valrojo | 10M/10F | 0.391 | 0.065 | 1.000 | 1.000 | 5.40 | 0.18 | 10 |
| 5 | Vegalatrave | 10M/10F | 0.503 | 0.072 | 0.996 | 1.000 | 4.40 | 0.18 | 11 |
| 96 | Padul | 10M/10F | 0.262 | 0.069 | 0.934 | 1.000 | 7.50 | 0.18 | 17 |
| 97 | LLano de Los Brincos-La Mota | 10M/10F | 0.177 | 0.057 | 0.966 | 1.000 | 6.90 | 0.17 | 14 |
| 98 | Los Sebastianes | 10M/10F | -0.029 | 0.054 | 0.240 | 0.994 | 12.50 | 0.24 | 22 |
| 99 | Karst de Sorbas | 10M/10F | 0.230 | 0.061 | 0.852 | 1.000 | 8.10 | 0.19 | 16 |
| 2 | Donadillo | 10M/6F | 0.255 | 0.058 | 0.992 | 1.000 | 5.70 | 0.17 | 13 |
| 3 | Villanueva de Valrojo | 10M/6F | 0.334 | 0.060 | 0.998 | 1.000 | 5.20 | 0.18 | 11 |
| 5 | Vegalatrave | 10M/6F | 0.456 | 0.066 | 1.000 | 1.000 | 4.10 | 0.17 | 10 |
| 96 | Padul | 10M/6F | 0.198 | 0.063 | 0.910 | 1.000 | 7.70 | 0.18 | 14 |
| 97 | LLano de Los Brincos-La Mota | 10M/6F | 0.137 | 0.051 | 0.958 | 1.000 | 6.80 | 0.18 | 15 |
| 98 | Los Sebastianes | 10M/6F | -0.028 | 0.053 | 0.342 | 1.000 | 11.80 | 0.23 | 19 |
| 99 | Karst de Sorbas | 10M/6F | 0.183 | 0.055 | 0.878 | 1.000 | 7.90 | 0.18 | 16 |

**Supplementary Table S7.** Effects of the translocation program in the metapopulation overall values. The simulations were carried out in two scenarios of recipient subpopulations (close and distant to the metapopulation core) and six alternatives of number of individuals moved: 1+1, 2+2, 3+3, 5+5, 10+10 and 10+6 males/females.

| **Scenario** | | ***r*** | ***SD(r)*** | ***CI(r)*** | ***N_10_*** | ***SD(N_10_)*** | ***CI(N_10_)*** | ***P_0_(10)*** | ***N_20_*** | ***SD(N_20_)*** | ***CI(N_20_)*** | ***P_0_(20)*** | ***T_med_*** | ***T_mean_*** | ***SD(T_mean_)*** | ***CI(T_mean_)*** | ***T_max_*** |
| --- | --- | --- | --- | --- | --- | --- | --- | --- | --- | --- | --- | --- | --- | --- | --- | --- | --- |
| No translocation | | -0.401 | 0.246 | 0.022 | 311.520 | 198.480 | 17.398 | 0.000 | 8.060 | 6.110 | 0.536 | 0.842 | 18 | 19.07 | 2.50 | 0.219 | 27 |
| 7 receipients close to the core | |  |  |  |  |  |  |  |  |  |  |  |  |  |  |  |  |
|  | 1M/1F | -0.405 | 0.247 | 0.022 | 290.730 | 169.320 | 14.842 | 0.000 | 0.920 | 2.190 | 0.192 | 0.890 | 18 | 18.93 | 2.13 | 0.187 | 24 |
|  | 2M/2F | -0.403 | 0.251 | 0.022 | 299.390 | 186.350 | 16.334 | 0.000 | 1.330 | 4.070 | 0.357 | 0.876 | 18 | 19.00 | 2.29 | 0.201 | 27 |
|  | 3M/3F | -0.407 | 0.251 | 0.022 | 289.540 | 174.310 | 15.279 | 0.000 | 9.700 | 16.640 | 1.459 | 0.886 | 18 | 18.74 | 2.26 | 0.198 | 27 |
|  | 5M/5F | -0.408 | 0.255 | 0.022 | 304.010 | 202.910 | 17.786 | 0.000 | 9.160 | 8.950 | 0.785 | 0.854 | 18 | 18.94 | 2.43 | 0.213 | 27 |
|  | 10M/10F | -0.411 | 0.254 | 0.022 | 270.200 | 174.630 | 15.307 | 0.000 | 5.880 | 6.470 | 0.567 | 0.904 | 18 | 18.63 | 2.15 | 0.188 | 25 |
|  | 10M/6F | -0.410 | 0.253 | 0.022 | 278.240 | 182.540 | 16.000 | 0.000 | 8.210 | 7.740 | 0.678 | 0.894 | 17 | 18.69 | 2.39 | 0.209 | 27 |
| 7 receipients distant to the core | | |  |  |  |  |  |  |  |  |  |  |  |  |  |  |  |
|  | 1M/1F | -0.404 | 0.249 | 0.022 | 303.840 | 182.470 | 15.994 | 0.000 | 7.130 | 5.470 | 0.479 | 0.866 | 18 | 19.00 | 2.25 | 0.197 | 26 |
|  | 2M/2F | -0.403 | 0.248 | 0.022 | 436.230 | 244.490 | 21.430 | 0.000 | 8.310 | 8.600 | 0.754 | 0.876 | 18 | 19.00 | 2.29 | 0.201 | 27 |
|  | 3M/3F | -0.401 | 0.251 | 0.022 | 302.100 | 196.610 | 17.234 | 0.000 | 7.940 | 8.200 | 0.719 | 0.856 | 18 | 19.03 | 2.36 | 0.207 | 27 |
|  | 5M/5F | -0.409 | 0.252 | 0.022 | 281.250 | 184.370 | 16.161 | 0.000 | 6.530 | 5.200 | 0.456 | 0.872 | 17 | 18.80 | 2.30 | 0.207 | 26 |
|  | 10M/10F | -0.408 | 0.246 | 0.022 | 253.030 | 166.350 | 14.581 | 0.000 | 7.050 | 6.720 | 0.589 | 0.880 | 18 | 18.69 | 2.40 | 0.207 | 27 |
|  | 10M/6F | -0.403 | 0.252 | 0.022 | 286.050 | 181.090 | 15.873 | 0.000 | 10.220 | 16.100 | 1.411 | 0.882 | 18 | 18.90 | 2.27 | 0.207 | 27 |

**Supplementary Table S8.** Selection of the land uses preferred by Dupont’s Lark. We intersected the 16,676 observations of the period 2000-2018 with the CORINE layers of years 2000, 2006, 2012 and 2018 and assigned to each point the most updated land category according to the date of the observation. The categories that accounted for 95% of the observations were interpreted as the preferred habitat of the species: 323, 321, 333 and 211.

| CORINE category | Nº of observations | % of total obs. | Cumulated % | Selected |
| --- | --- | --- | --- | --- |
| 323 | 8121 | 48.70 | 48.70 | X |
| 321 | 6332 | 37.97 | 86.67 | X |
| 333 | 743 | 4.46 | 91.12 | X |
| 211 | 656 | 3.93 | 95.06 | X |
| 243 | 612 | 3.67 | 98.73 |  |
| 311 | 82 | 0.49 | 99.22 |  |
| 324 | 45 | 0.27 | 99.49 |  |
| 334 | 26 | 0.16 | 99.65 |  |
| 231 | 16 | 0.10 | 99.74 |  |
| 322 | 12 | 0.07 | 99.81 |  |
| 242 | 11 | 0.07 | 99.88 |  |
| 312 | 9 | 0.05 | 99.93 |  |
| 411 | 7 | 0.04 | 99.98 |  |
| 222 | 2 | 0.01 | 99.99 |  |
| 212 | 1 | 0.01 | 99.99 |  |
| 313 | 1 | 0.01 | 100.00 |  |


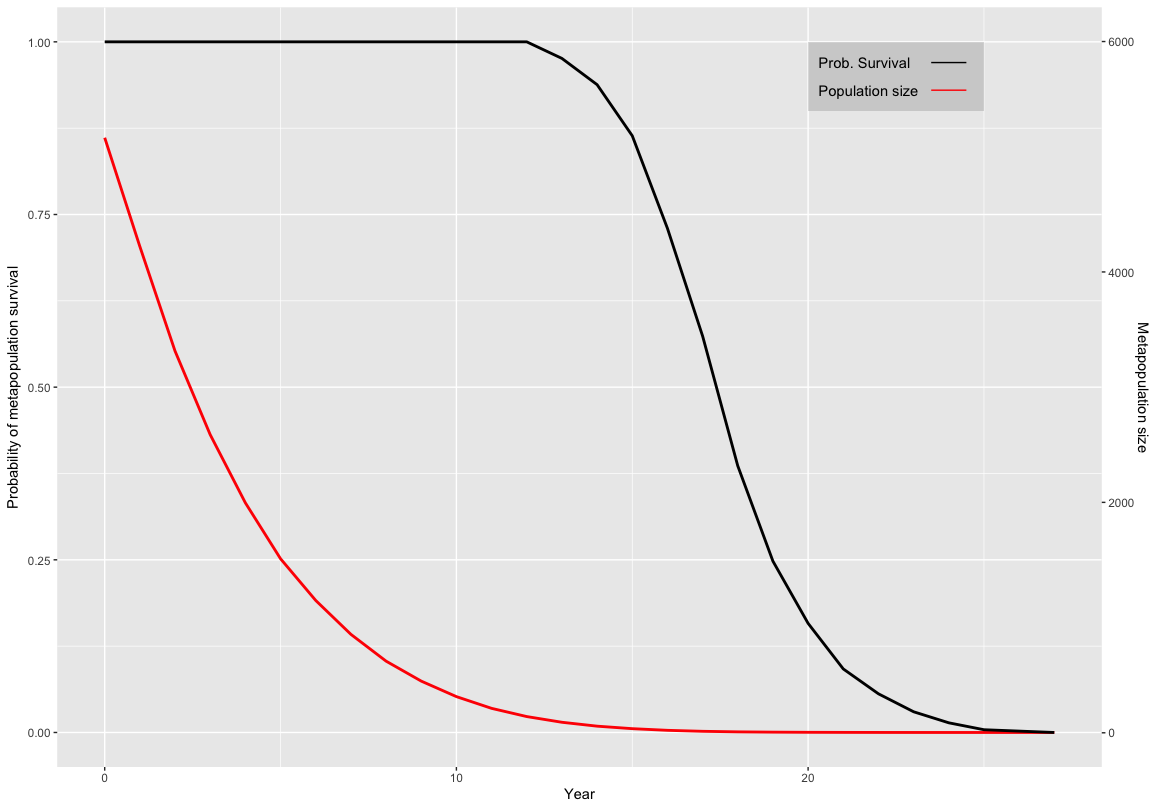


**Supplementary Figure S1**. Results of the PVA base model of Dupont’s Lark metapopulation with 500 iterations.

**
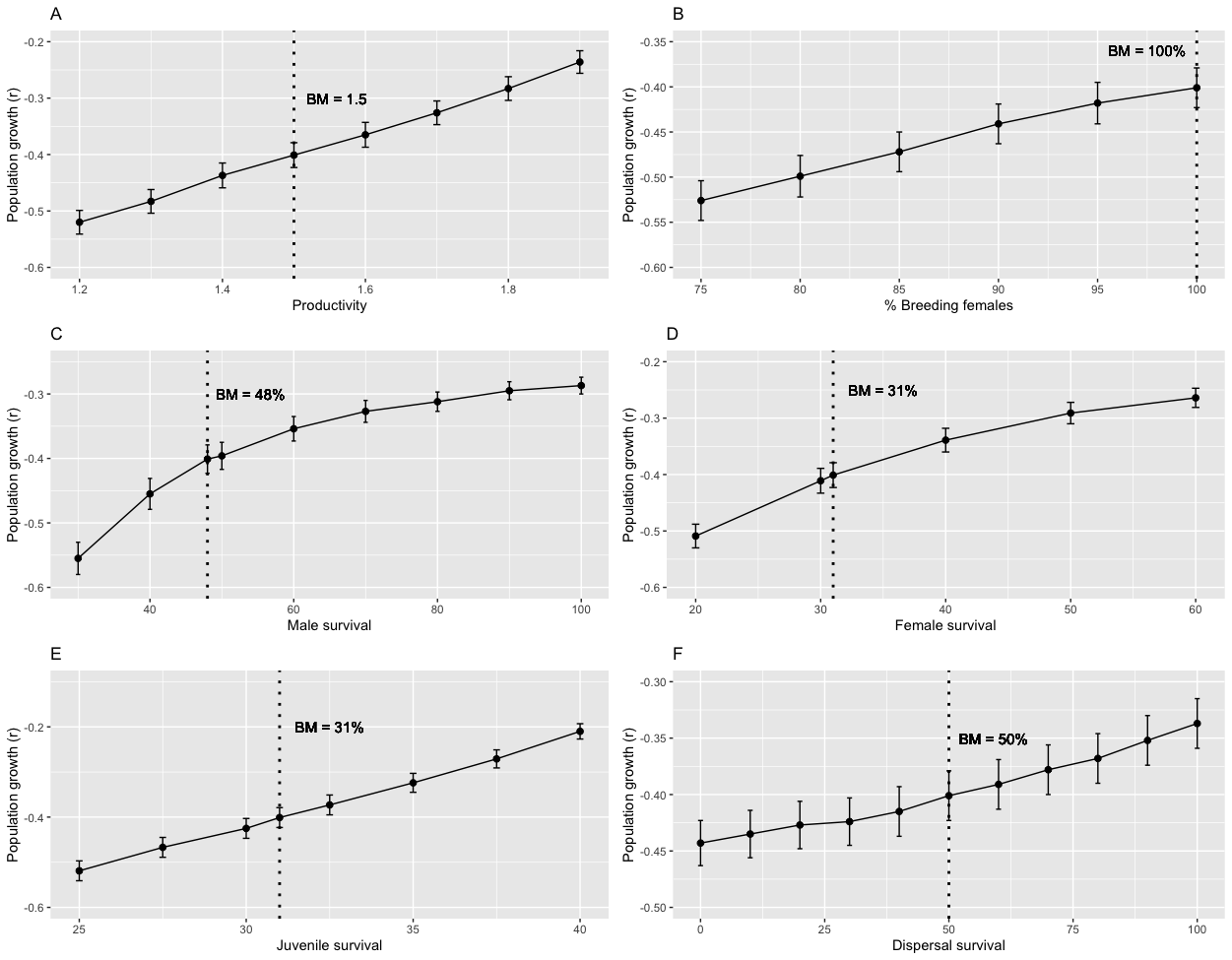
**

**Supplementary Figure S2**. Effects of the parameters evaluated in the sensitivity analysis on the population growth (mean *r* value and 95% confidence interval). Dotted lines represent the parameter value in the base model. A: productivity (offspring/brood); B: % of breeding females; C: adult male survival; D: adult female survival; E: juvenile survival (both sexes); F: survival of dispersers.

**
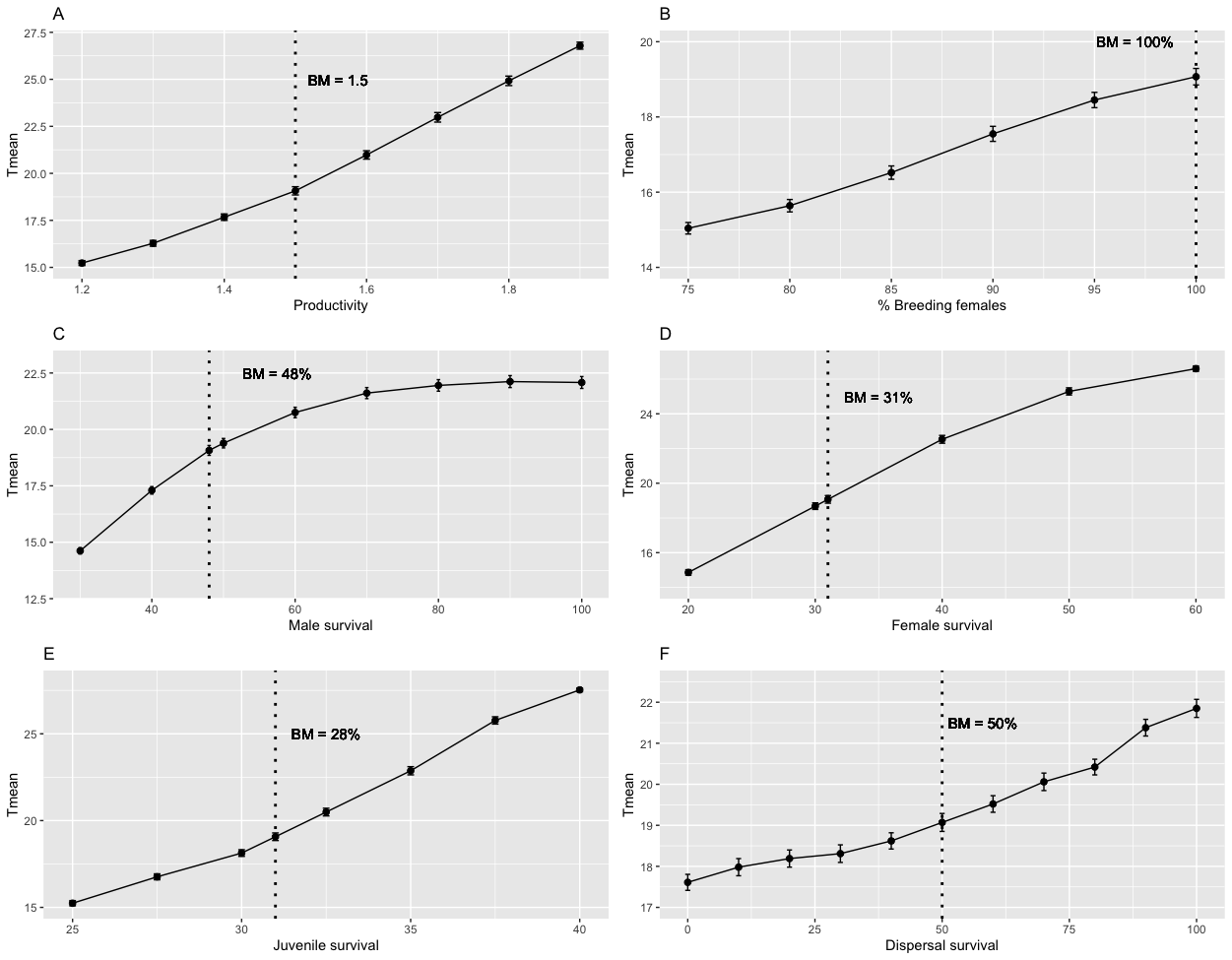
**

**Supplementary Figure S3**. Effects of the parameters evaluated in the sensitivity analysis on the mean time to extinction (*T_mean_* and 95% confidence interval). Dotted lines represent the parameter value in the base model. A: productivity (offspring/brood); B: % of breeding females; C: adult male survival; D: adult female survival; E: juvenile survival (both sexes); F: survival of dispersers.

**
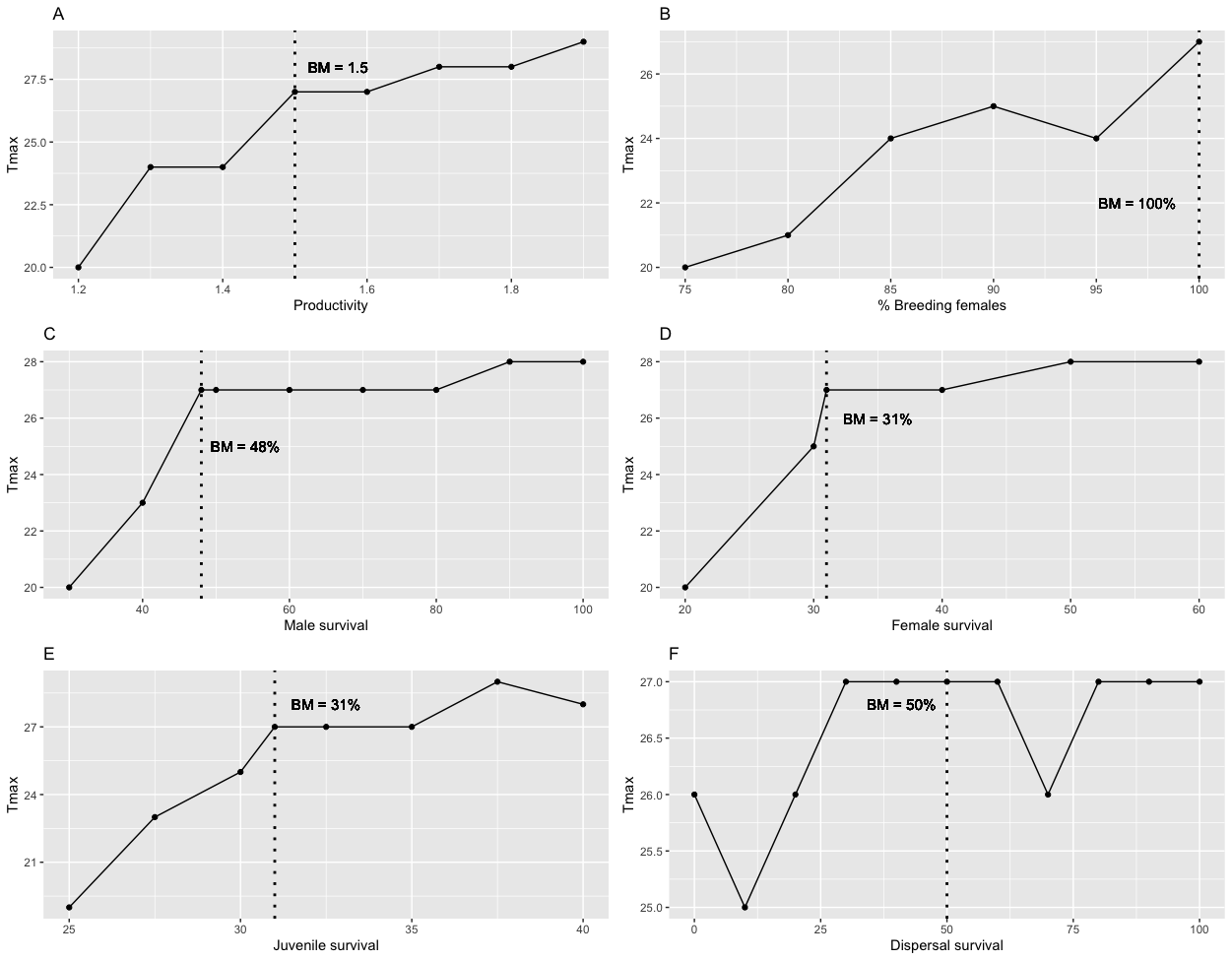
**

**Supplementary Figure S4**. Effects of the parameters evaluated in the sensitivity analysis on the maximum time to extinction (*T_max_*). Dotted lines represent the parameter value in the base model. A: productivity (offspring/brood); B: % of breeding females; C: adult male survival; D: adult female survival; E: juvenile survival (both sexes); F: survival of dispersers.


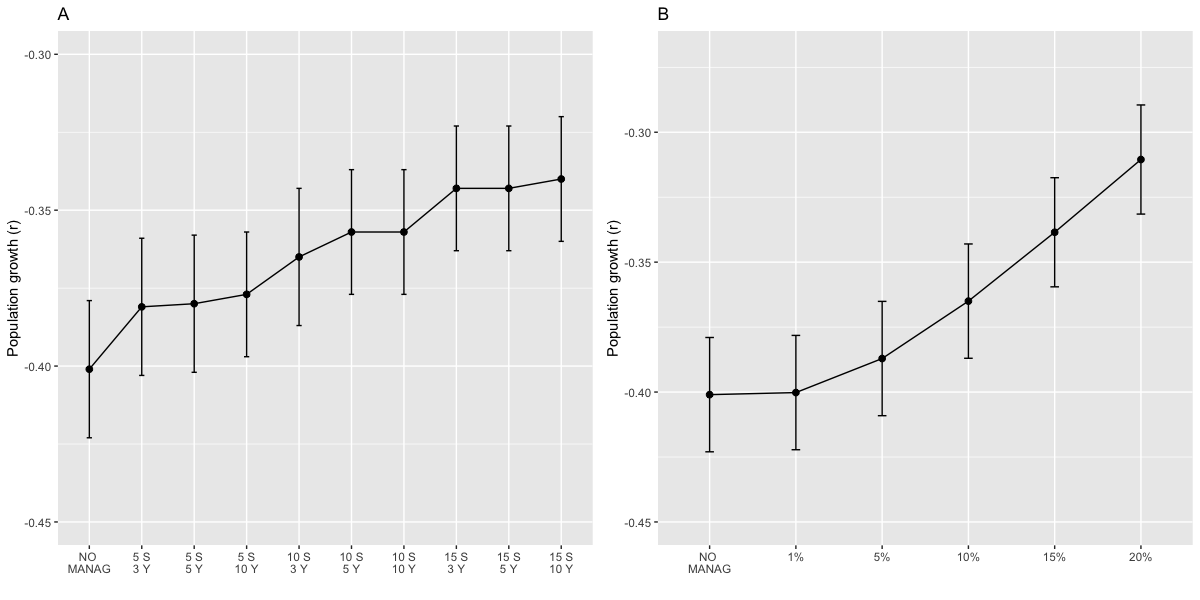


**Supplementary Figure S5**. Effects of habitat restoration simulations in metapopulation growth rate. S5A: nine different scenarios varying the number of subpopulations managed (selecting those with higher population size: 5, 10 and 15 subpopulations) and time period of the program (3, 5 and 10 consecutive years). S5B: using the alternative of 10 subpopulations during 3 years of management (considered of real applicability), five scenarios of management intensity were tested (1, 5, 10, 15 and 20% increase of productivity and carrying capacity).


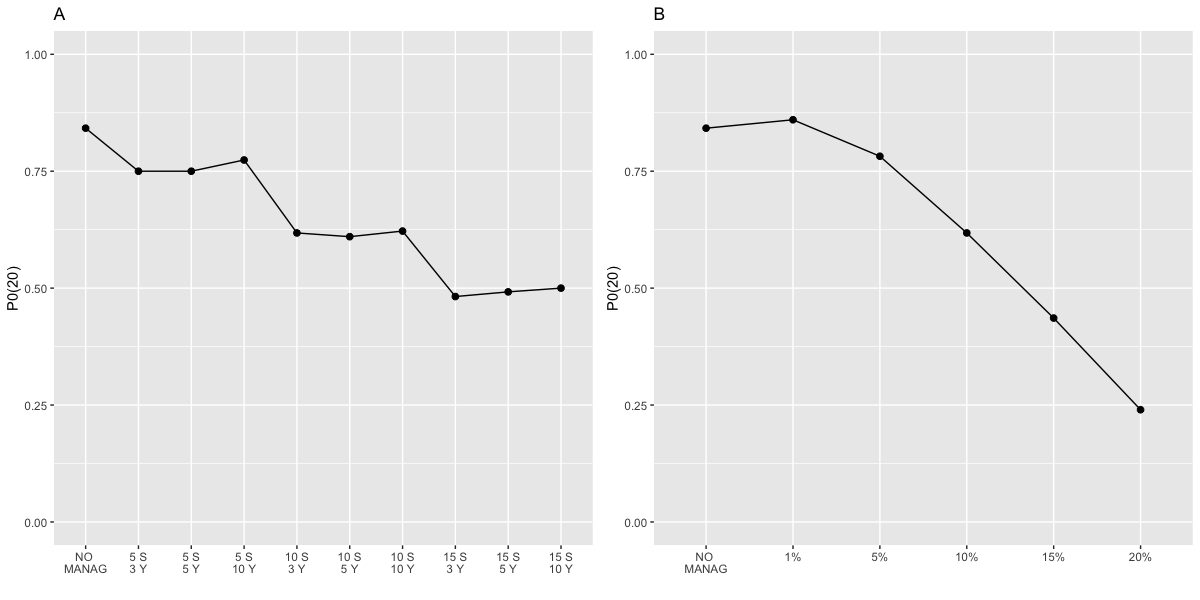


**Supplementary Figure S6**. Effects of habitat restoration simulations in metapopulation probability of extinction in 20 years. S6A: nine different scenarios varying the number of subpopulations managed (selecting those with higher population size: 5, 10 and 15 subpopulations) and time period of the program (3, 5 and 10 consecutive years). S6B: using the alternative of 10 subpopulations during 3 years of management (considered of real applicability), five scenarios of management intensity were tested (1, 5, 10, 15 and 20% increase of productivity and carrying capacity).


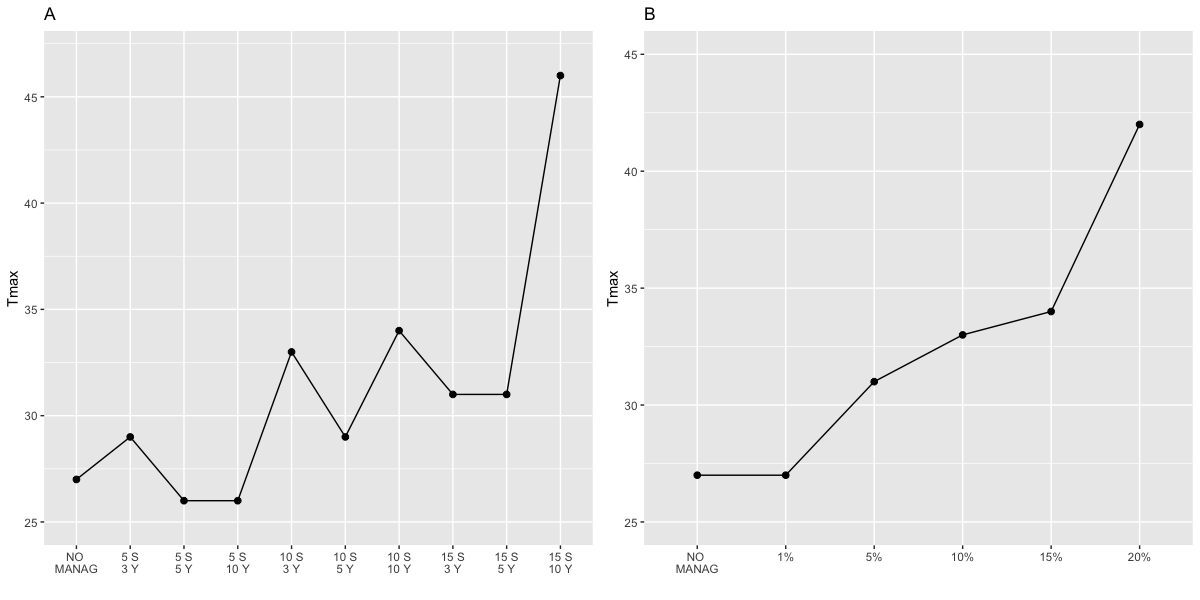


**Supplementary Figure S7**. Effects of habitat restoration simulations in metapopulation maximum time to extinction. S7A: nine different scenarios varying the number of subpopulations managed (selecting those with higher population size: 5, 10 and 15 subpopulations) and time period of the program (3, 5 and 10 consecutive years). S7B: using the alternative of 10 subpopulations during 3 years of management (considered of real applicability), five scenarios of management intensity were tested (1, 5, 10, 15 and 20% increase of productivity and carrying capacity).


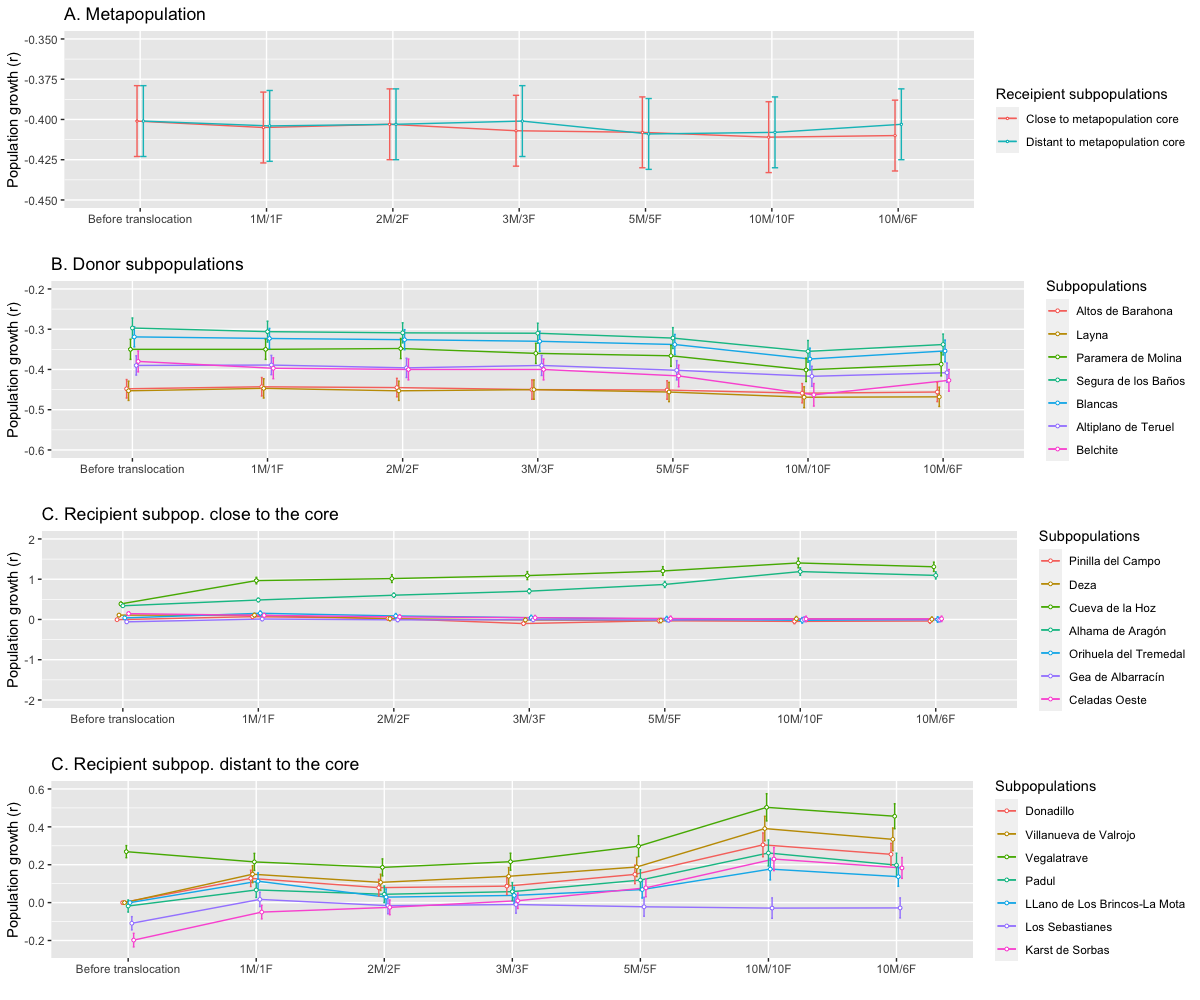


**Supplementary Figure S8**. Effects in growth rate (*r*) (*r* and 95% confidence interval) in the metapopulation, donor and recipient subpopulations after the translocation program. Donor subpopulations were those with more than 100 males (n=7). Recipient subpopulations were those with the most unfavorable situation in the PVA results (shortest mean time to extinction), two scenarios were simulated: seven recipient subpopulations close to the metapopulation core and seven recipients distant to the core. For each scenario, six different harvest alternatives are offered: 1+1, 2+2, 3+3, 5+5, 10+10 and 10+6 males/females, this last one adjusted to the species sex ratio. In all of them, movements were carried out during 3 consecutive years. Harvested individuals were introduced randomly in recipient subpopulations (Supplementary Table S6).


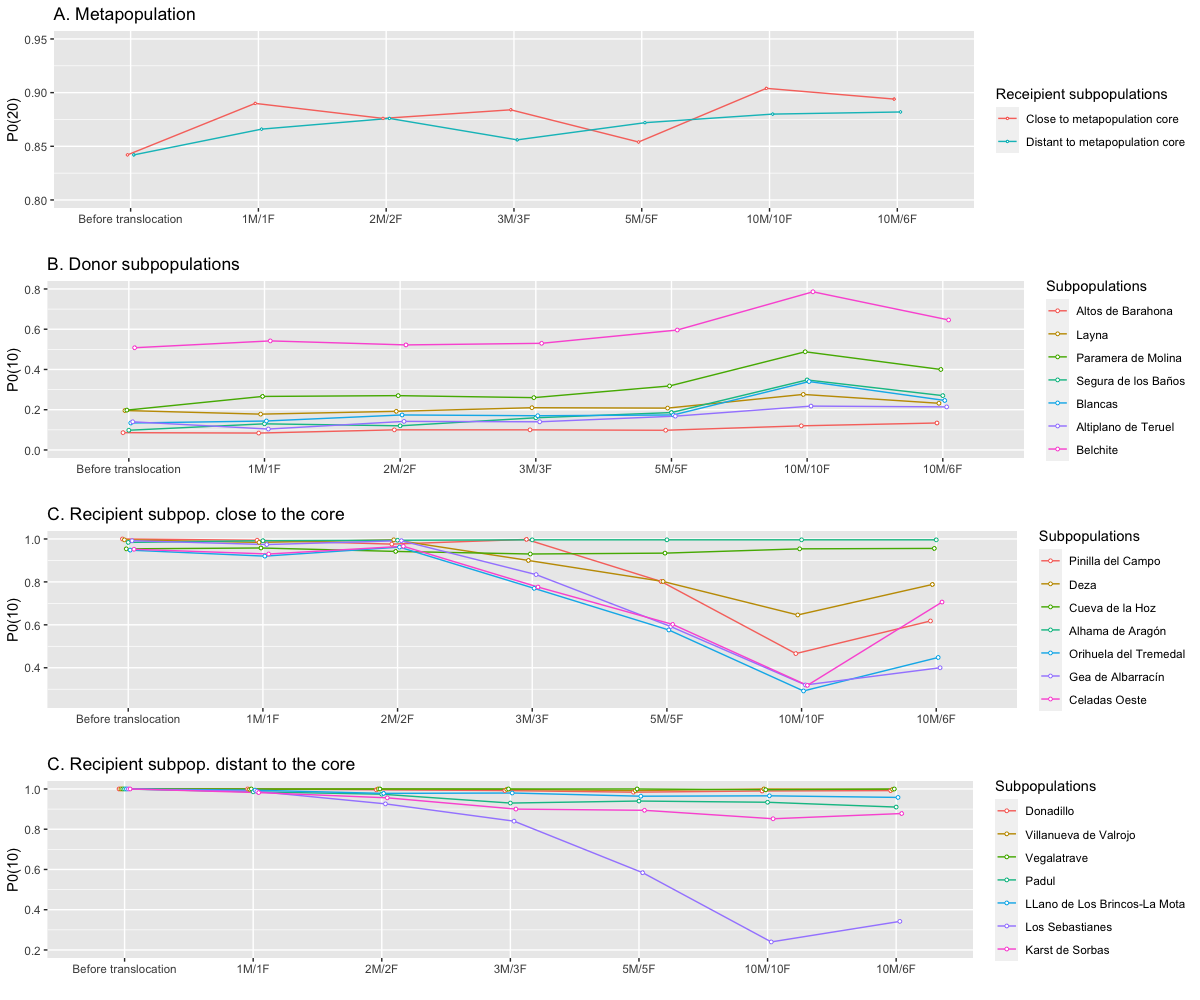


**Supplementary Figure S9**. Effects in probability of extinction (*P_0_(20)* for metapopulation and *P_0_(10)* for donor and recipient subpopulations, as most of them reach extinction before year 20) after the translocation program. Donor subpopulations were those with more than 100 males (n=7). Recipient subpopulations were those with the most unfavorable situation in the PVA results (shortest mean time to extinction), two scenarios were simulated: seven recipient subpopulations close to the metapopulation core and seven recipients distant to the core. For each scenario, six different harvest alternatives are offered: 1+1, 2+2, 3+3, 5+5, 10+10 and 10+6 males/females, this last one adjusted to the species sex ratio. In all of them. movements were carried out during 3 consecutive years. Harvested individuals were introduced randomly in recipient subpopulations (Supplementary Table S6).


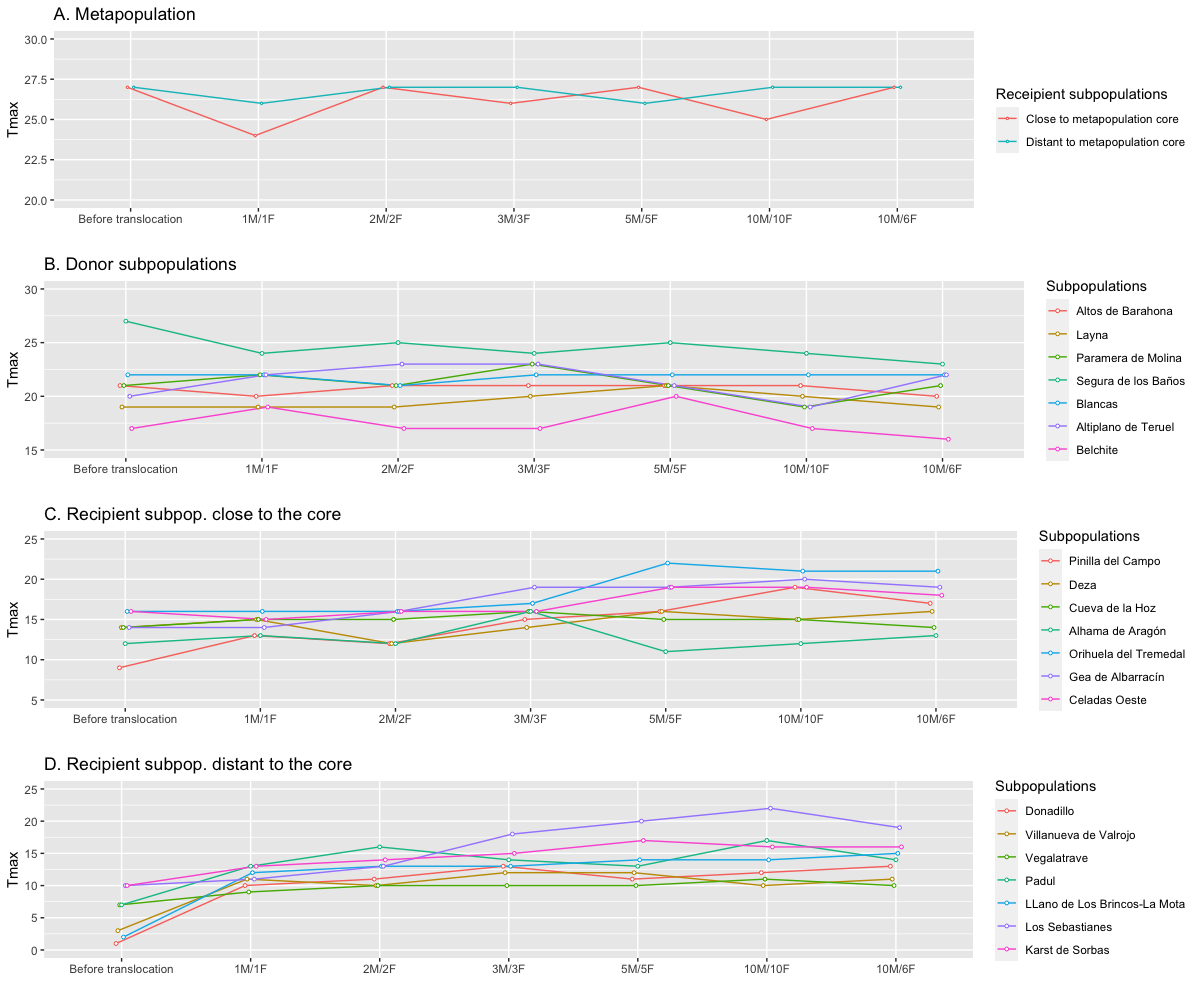


**Supplementary Figure S10**. Effects in maximum time to extinction in the metapopulation, donor and recipient subpopulations. Donor subpopulations were those with more than 100 males (n=7). Recipient subpopulations were those with the most unfavorable situation in the PVA results (shortest mean time to extinction), two scenarios were simulated: seven recipient subpopulations close to the metapopulation core and seven recipients distant to the core. For each scenario, six different harvest alternatives are offered: 1+1, 2+2, 3+3, 5+5, 10+10 and 10+6 males/females, this last one adjusted to the species sex ratio. In all of them, movements were carried out during 3 consecutive years. Harvested individuals were introduced randomly in recipient subpopulations (Supplementary Table S6).
